# Supplementary material for: Ribosome profiling reveals a functional role for autophagy in mRNA translational control
Source: Commun Biol. 2020 Jul 17;3:388. doi: 10.1038/s42003-020-1090-2 (PMC7367890; doi:10.1038/s42003-020-1090-2)

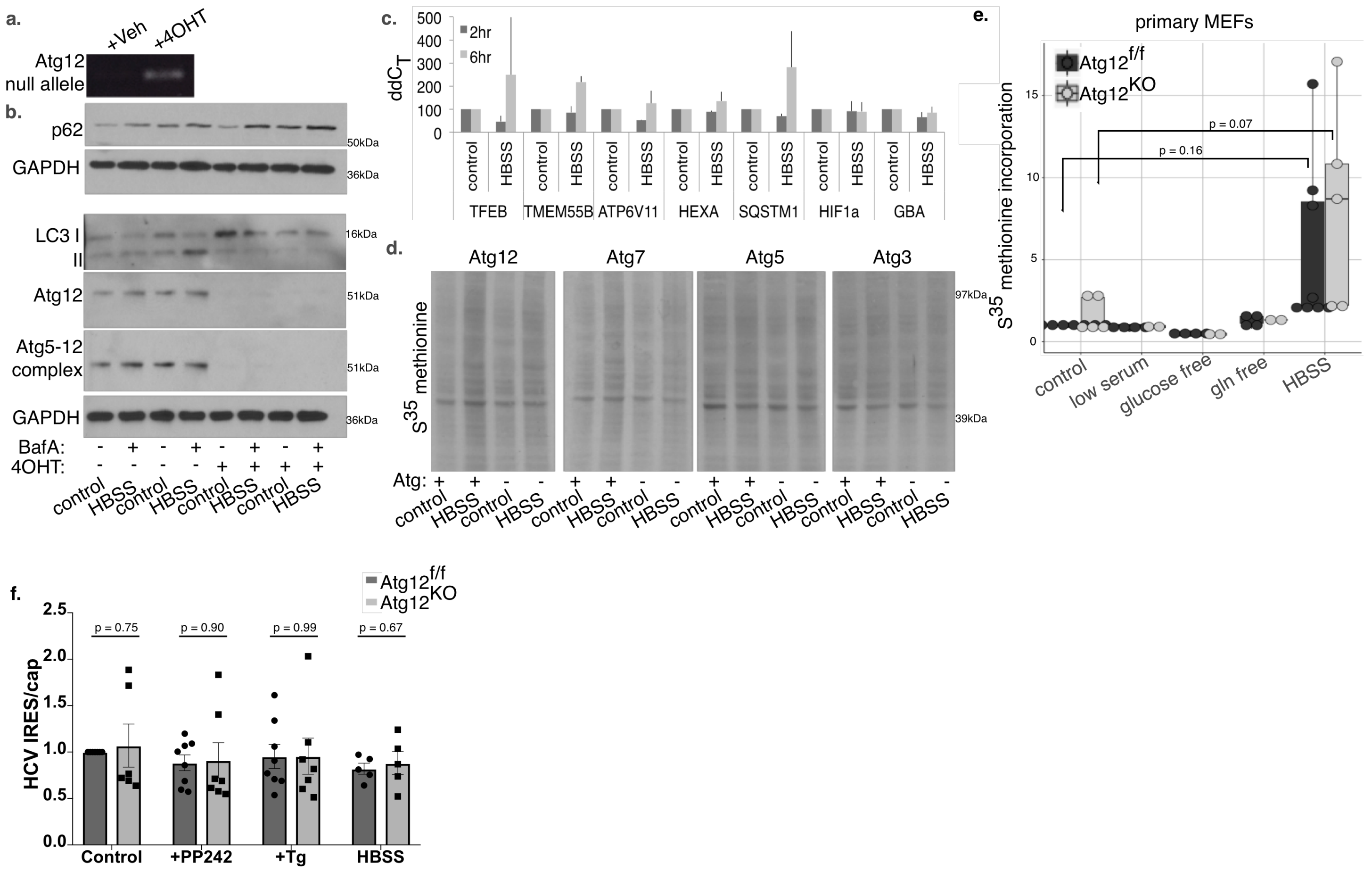

Supplementary Figure 1: Analysis of Atg deletion in mouse embryonic fibroblasts on translation rate (related to Figure 1)

a. PCR of the null allele of Atg12 in immortalized Atg12$^{f/f}$ MEFs at 2d following treatment with vehicle control or 4OHT (20µM).

b. Protein lysate collected from Atg12$^{f/f}$ MEFs five days following treatment with vehicle control or 4OHT (20µM). Indicated cells were incubated with fresh control (nutrient rich) media, or HBSS starved for 2h. When indicated, Bafilomycin A (Baf A, 2.5ng/ml) was added at 30min before lysis. Lysates were immunoblotted as indicated.

c. Atg12$^{f/f}$ MEFs were grown in control media or subject to 2 or 6 hours of HBSS starvation. Quantification (mean ± SD, n=3 biologically independent replicates) of percent expression of various genes (as indicated) by qPCR with GAPDH as endogenous control is graphed. t tests were

performed on the linearized Ct normalized to GAPDH levels for each biological replicate, and there were no significant changes ($p > 0.05$) between control and HBSS treated at two hours for each gene.

d. Representative ^{35}S-methionine incorporation autoradiogram for immortalized MEFs derived from mice genetically deleted for the indicated Atgs essential for autophagy. Cells were labeled and lysed in either methionine-free control conditions, or following 2h HBSS starvation.

e. Primary Atg12$^{f/f}$ or Atg12^{KO} MEFs were grown in control media, and two hours prior to lysis were switched to control media lacking methionine, low (1%) serum media lacking methionine, media lacking glucose and methionine, media lacking glutamine and methionine, or HBSS. Cells were labeled with ^{35}S-methionine for 30 min prior to lysis. The relative ^{35}S-methionine incorporation rates is quantified and shown is a boxplot with dotplot overlay for each biological replicate, normalized to loading control. ANOVA with Tukey's post hoc test were performed on the data, p values between control and HBSS for Atg12$^{f/f}$ or Atg12^{KO} are shown.

f. Quantification (mean ± SEM, n=5, p-value by t test) of renilla luciferase activity driven by the Hepatitis C viral IRES motif, normalized to firefly luciferase activity driven by the cap. Luminescence was assayed using Dual-Glo reagents (Promega). PP242 (2µM for1h) inhibits mTORC1 signaling, and thapsigargin (Tg, 1µM for 1h) induces IRES mediated translation.

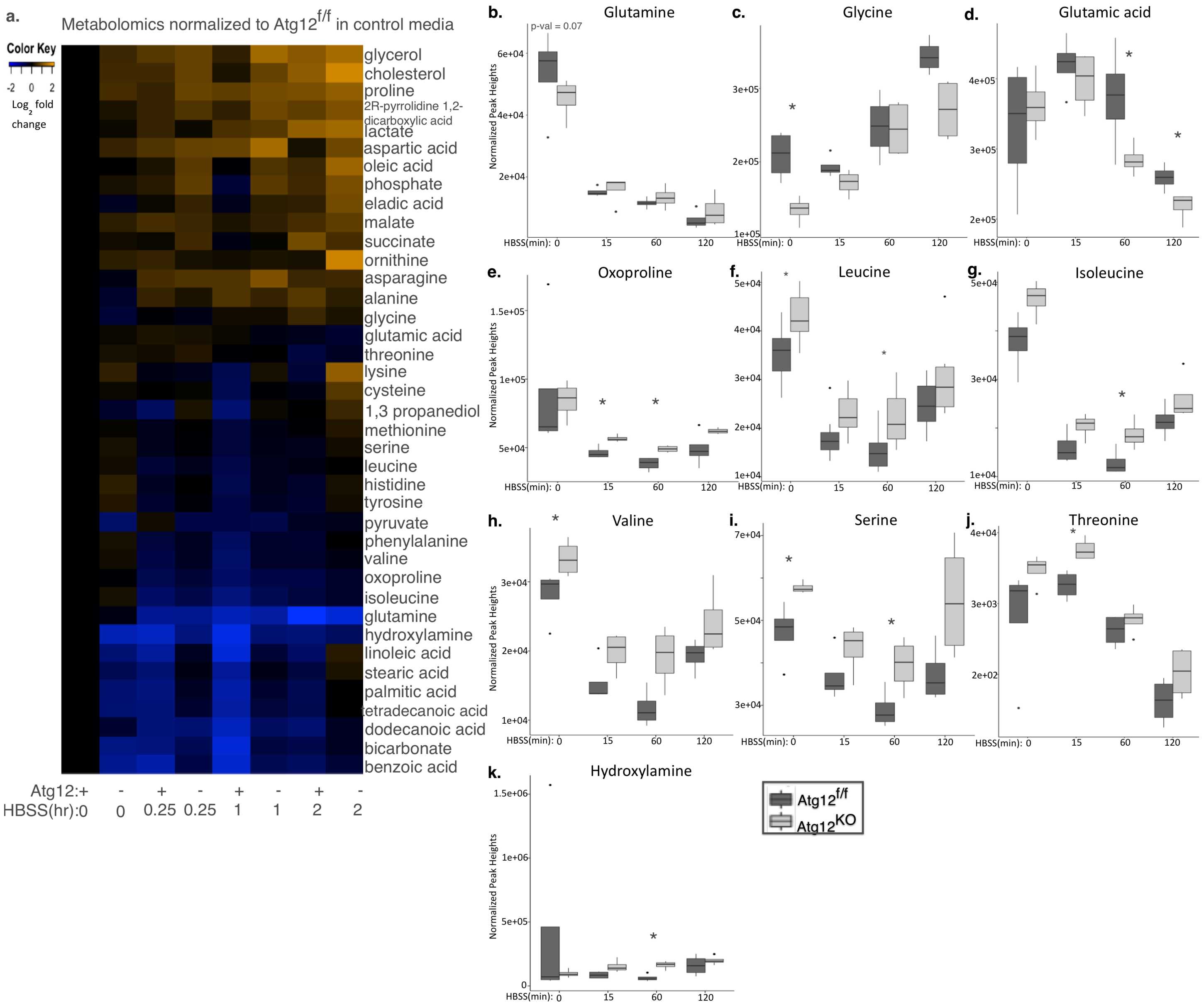

Supplementary Figure 2: Amino acid levels in Atg12^{KO} MEFs (related to Figure 2)

a. Changes in intracellular metabolite levels detected by GC-TOF with MTBSTFA (n=4), in Atg12$^{f/f}$ and Atg12^{KO} MEFs in control media or following HBSS starvation for the indicated times. Fold change is relative to Atg12$^{f/f}$ in control media.

b-k. Normalized peak height data for individual metabolites from metabolomics profiling using gas chromatography time of flight mass spectrometry with the silylation reagent N-tert-butyldimethylsilyl-N-methyltrifluoroacetamide (GC-TOF with MTBSTFA). Boxplots of normalized peak height of each metabolite as indicated, in Atg12$^{f/f}$ or Atg12^{KO} MEFs grown in control media (t=0), or HBSS starved for the indicated times. Asterisks indicate a $p < 0.05$ by t test.

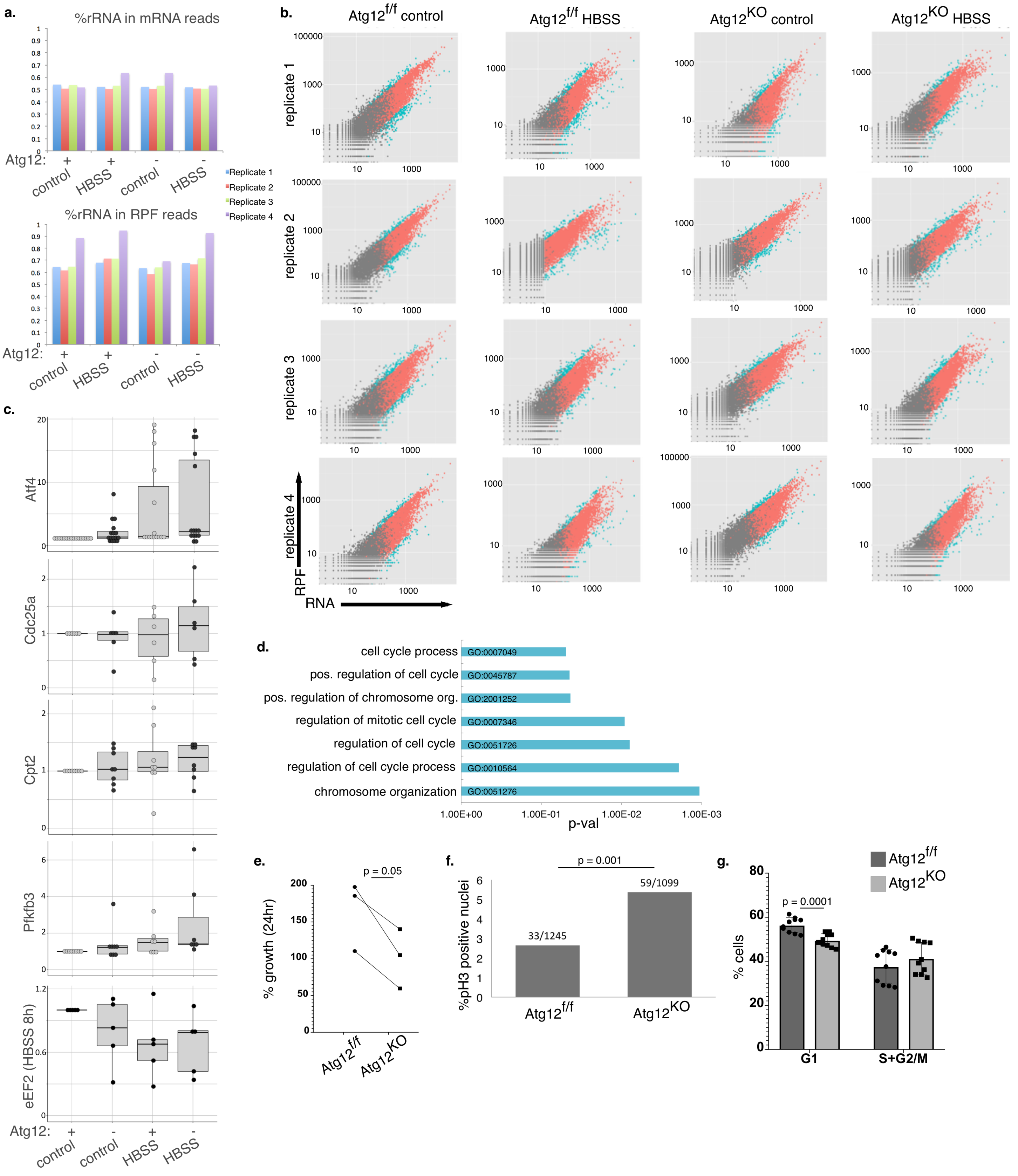

Supplementary Figure 3: Ribosome profiling quality control metrics (related to Figure 3)

a. Graph of percent of contaminating rRNA reads in total RNA samples and RPF samples from each biological replicate that were removed in Babel processing.

b. Read counts of RPF by RNA per condition per biological replicate. Blue dots are genes with one-sided p-value <0.025 corresponding to increased or decreased RPF, red dots are genes with one-sided p-value between 0.025 and 0.975, grey dots indicate one-sided p-value = NA.

c. Quantification of immunoblot band intensity, normalized to control, for significant hits from RP analysis are shown by boxplots, with dotplot overlay for each biological replicate. Prior to lysis, Atg12$^{f/f}$ or Atg12^{KO} MEFs were maintained in control media, or starved in HBSS for 2 hours, unless indicated for 8 hours (last panel, eEF2). No change was statistically significant ($p > 0.05$) between Atg12$^{f/f}$ or Atg12^{KO} MEFs by ANOVA with Tukey's HSD post hoc test.

d. Significantly enriched biological processes based on gene ontology (GO) analysis of mRNAs whose ribosome occupancy is significantly decreased in Atg12^{KO} versus Atg12$^{f/f}$ cells.

e. Quantification (mean ± SEM, n=3 biologically independent replicates) of percent growth of Atg12$^{f/f}$ and Atg12^{KO} MEFs over 24h measured by crystal violet staining. $p = 0.05$ by t test.

f. Percent of pH3 positive nuclei by immunofluorescence analysis in Atg12$^{f/f}$ and Atg12^{KO} MEFs. Fraction above bar indicates pH3 positive cells out of total number of cells enumerated from 3 biological replicates. $p = 0.001$ by two-sided fisher's exact test.

g. Cell cycle quantification (mean + SD, n=10 biologically independent replicates) of unsynchronized Atg12$^{f/f}$ or Atg12^{KO} MEFs by propidium iodide staining and flow cytometry. $p = 0.001$ by t test.

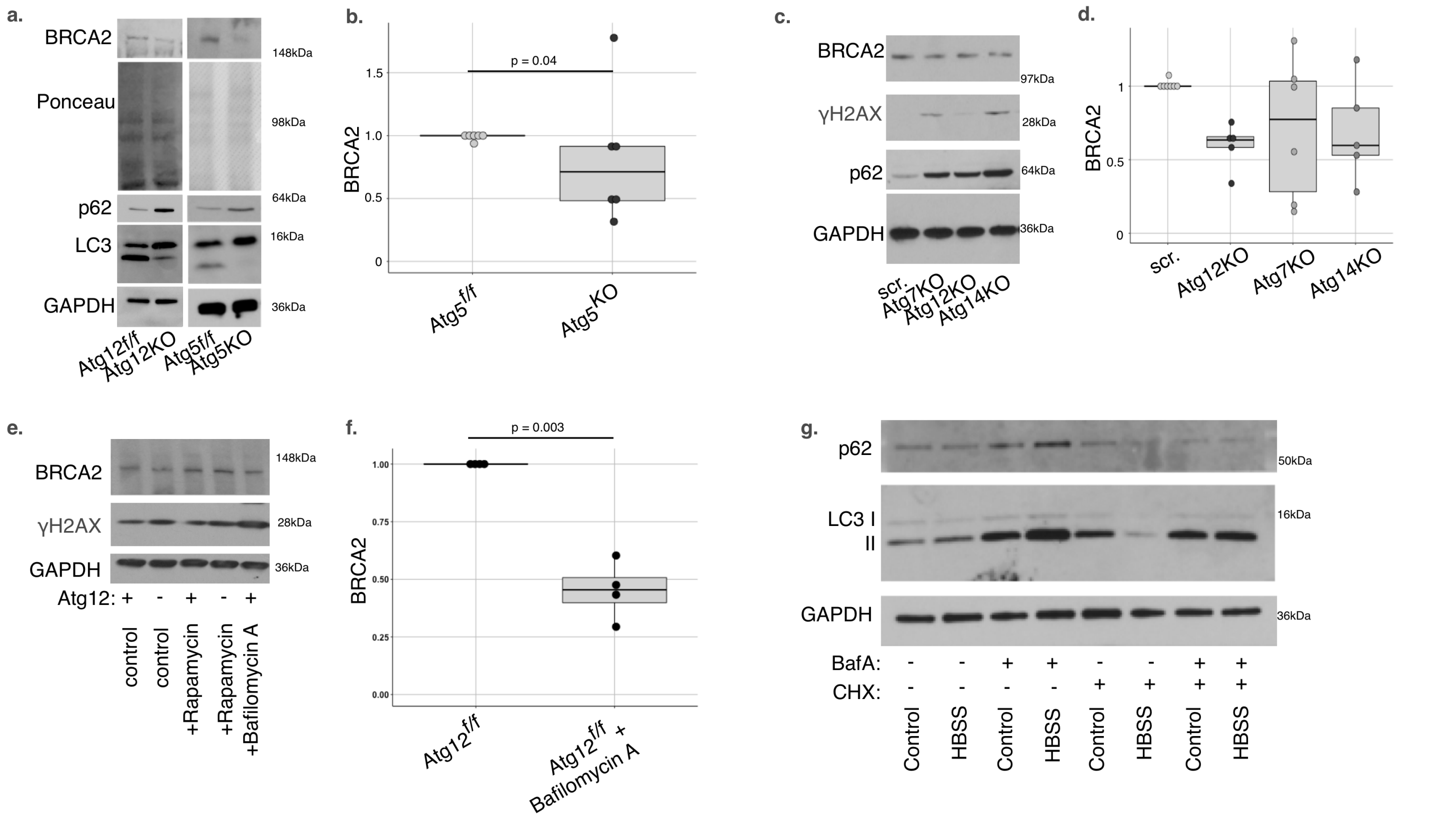

Supplementary Figure 4: *Brca2* translation is impaired in autophagy deficient cells (related to Figure 4)

a,b. Protein lysate from Atg12$^{f/f}$ and Atg12^{KO} MEFs, Atg5$^{f/f}$ and Atg5^{KO} MEFs was (**a**) immunoblotted as indicated and (**b**) relative BRCA2 levels normalized to loading control was quantified for each independent biological replicate. Statistical analysis was performed by t test.

c,d. Protein lysate was collected from HEK293T cells with CRISPR deleted Atg12, Atg7, Atg14, or a scrambled sgRNA control, and (**c**) immunoblotted for the indicated proteins; (**d**) relative BRCA2 protein levels normalized to loading control was quantified. Statistical analysis was performed by ANOVA with Tukey's HSD post hoc test; there was no significant difference between the control cells and the Atg deficient cells.

e,f. Protein lysate from Atg12$^{f/f}$ MEFs treated with control DMSO, 1uM Rapamycin or 20nM Bafilomycin A for 4h was collected and (**e**) immunoblotted as indicated. (**f**) relative BRCA2 levels, normalized to loading control, was quantified from control and Bafilomycin A treated MEFs. Statistical analysis was performed by t test.

g. Protein lysate was collected from MEFs treated with the lysosome inhibitor Bafilomycin A and cycloheximide (CHX) and immunoblotted for markers of autophagic turnover and loading controls as indicated.

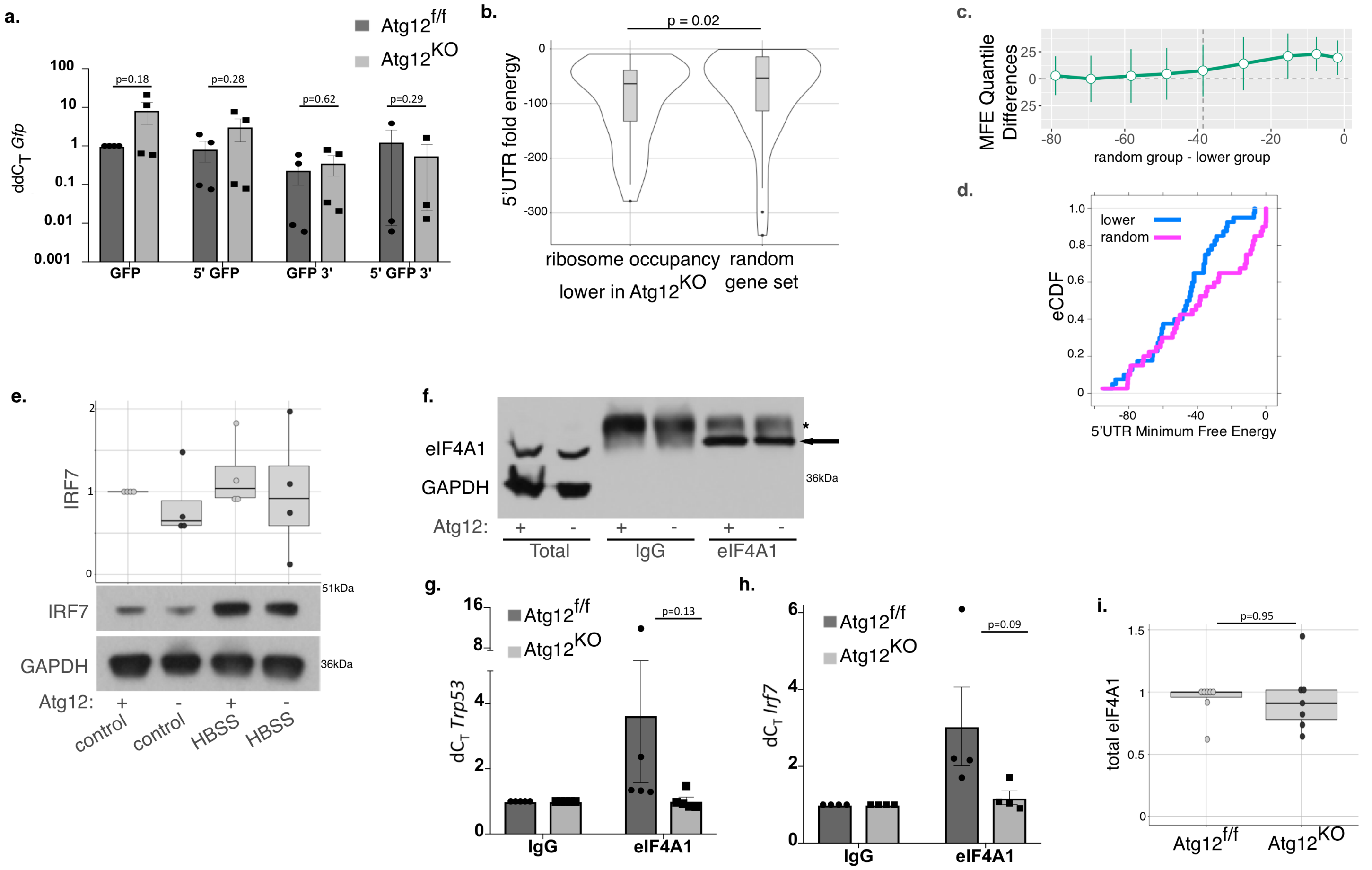

Supplementary Figure 5: The 5'UTR of *Brca2* determines its translational response to autophagy (related to Figure 5)

a. qPCR for relative levels of *Gfp* in Atg12$^{f/f}$ and Atg12^{KO} MEFs transfected with the pcDNA3 *Brca2* UTR reporter plasmids, using *Gapdh* as an endogenous control (mean ± SEM, minimum of 3 biologically independent replicates).

b. Plot of 5'UTR fold energies (boxplots on violin plots) from mRNAs with significantly lower than expected ribosome occupancy in Atg12^{KO} MEFs compared to a randomly generated gene set from the mouse genome of equivalent length. Statistical analysis performed using Wilcoxon rank sum test.

c,d. Local minimum free energy (MFE) was predicted by RNALfold in the 5'UTRs from mRNAs with significantly lower than expected ribosome occupancy in Atg12^{KO} MEFs (lower group) compared to a randomly generated gene set (random group). (**c**) The quantile differences between the MFEs of the two groups is graphed. (**d**) The empirical cumulative distribution function (eCDF) of the MFEs is plotted.

e. Protein lysate was collected from Atg12$^{f/f}$ or Atg12^{KO} MEFs grown in control media or starved in HBSS for 2h. IRF7 levels were measured by immunoblotting. Relative quantification from immunoblots for IRF7 normalized to loading control is shown in the boxplot with dotplot overlay per biological replicate. Bottom: representative immunoblot. An outlier (Dixon test $p = 0.02$) was excluded from the statistical analysis, $p < 0.001$ for Atg12$^{f/f}$ compared to Atg12^{KO} in control conditions by t test.

f. Representative immunoblot for eIF4A1 and GAPDH following RNA immunoprecipitation is shown. Arrow indicates eIF4A1, asterisk indicates immunoglobulin heavy chain.

g,h. Quantification (mean ± SD, n= minimum of 4 independent biological replicates) of the fold enrichment of (**g**) *Trp53* (t test, $p = 0.13$) and (**h**) *Irf7* (t test, $p = 0.09$) interaction with eIF4A1 over IgG control in Atg12$^{f/f}$ or Atg12^{KO} MEFs by RNA immunoprecipitation experiments.

i. Protein lysate was collected from Atg12$^{f/f}$ and Atg12^{KO} MEFs, and immunoblotted for total levels of eIF4A1. Relative levels normalized to loading control were quantified; a boxplot with dotplot overlay for each biological replicate is shown, $p = 0.95$ by t test.

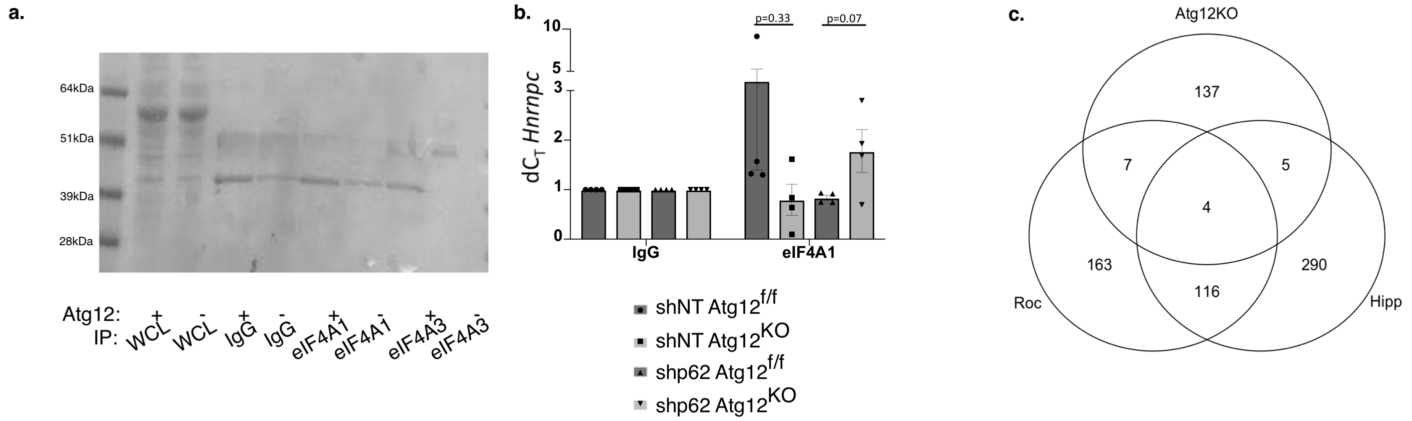

Supplementary Figure 6: eIF4A1 perturbations in Atg12^{KO} MEFs are unique compared to inhibition of eIF4A1 (related to Figure 6)

a. Representative total protein of eIF4A1 immunoprecipitation, measured by ponceau S stain.

b. Quantification (mean ± SEM, n=4 independent biological replicates) of the fold enrichment of *Hnrnpc* interaction with eIF4A1 over IgG control in Atg12$^{f/f}$ or Atg12^{KO} MEFs that were knocked down for p62/SQSTM1 or treated with non-targeting shRNA, assayed by RNA immunoprecipitation. p values were calculated by ANOVA with Tukey’s post hoc test.

c. Venn diagram showing overlap of mRNAs whose translation is sensitive (decreased ribosome occupancy) to autophagy inhibition (Atg12^{KO}), the eIF4A1 inhibitor Rocaglate A (Roc), and the eIF4A1 inhibitor Hippuristanol (Hipp) (data from Iwasaki et al, 2016).

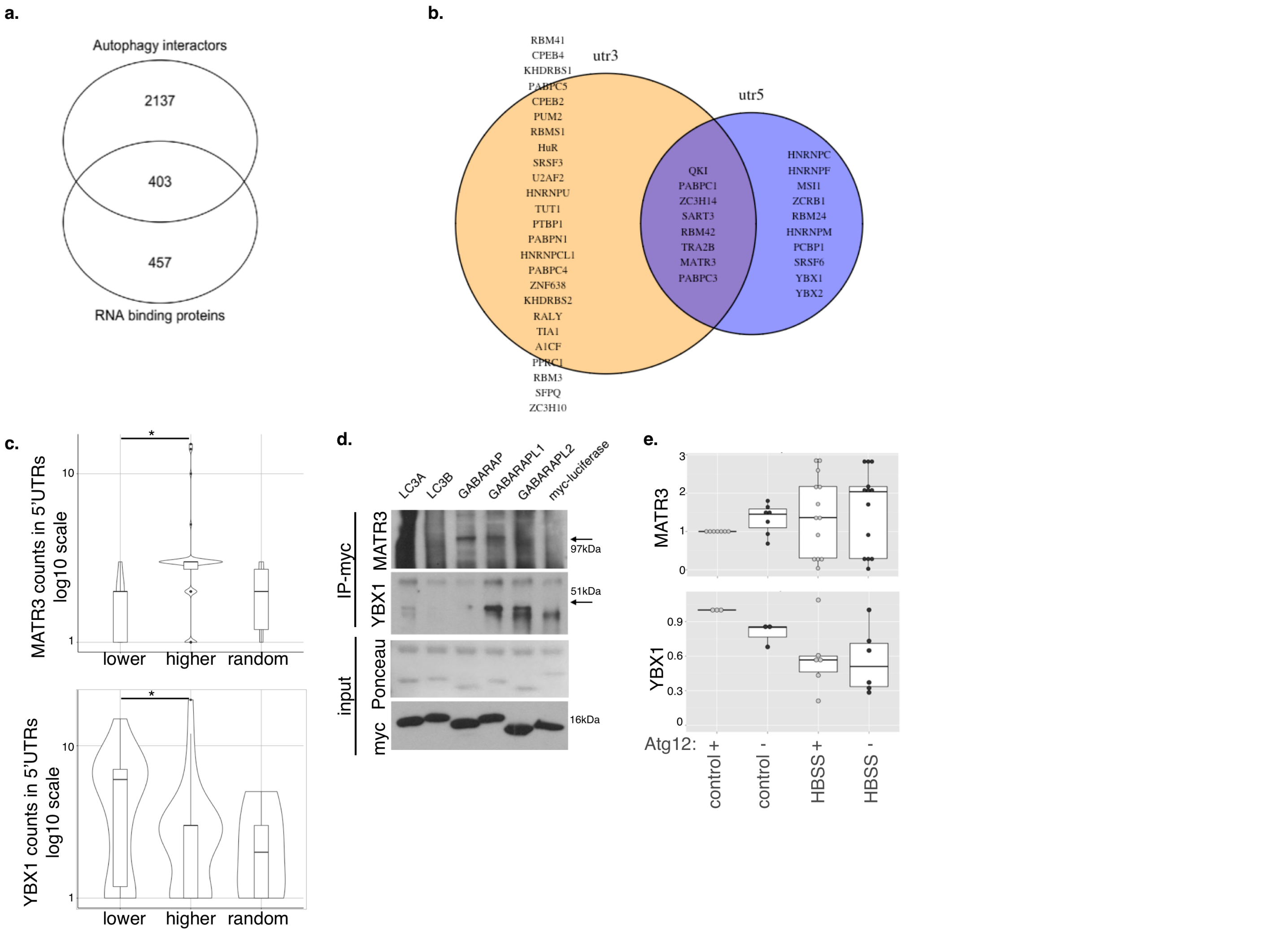

Supplementary Figure 7: Other RNA binding proteins are linked to autophagy enabled translation (related to Figure 7)

a. Analysis of overlap between data sets from Behrends et al, 2010 and Castello et al, 2012.

b. RBPDB was used to identify predicted RNA binding motifs in the UTR sequences of mRNAs with significantly altered ribosome occupancy, as described in Figure 2, and these groups with lower than expected ribosome occupancy and higher than expected ribosome occupancy were compared to each other and a group of UTR sequences from a randomly generated gene set of equivalent length. Significant differences in the count of RNA binding motifs were identified by Kruskal-Wallis test, represented in a Venn diagram, separated by UTR location.

c. The RBP motif count for MATR3 and YBX1 per 5'UTR in the gene sets for lower ribosome occupancy in Atg12^{KO} cells (lower), higher ribosome occupancy in Atg12^{KO} cells (higher), and a

randomly generated gene set (random) are plotted as a violin plot with a boxplot overlay. Asterisk indicates $p < 0.05$ by ANOVA with Tukey's HSD post hoc test.

d. Representative immunoblot of immunoprecipitation of myc-tagged overexpressed LC3 family members interaction with MATR3 and YBX1 in HEK293T cells.

e. Boxplot, with dotplot overlay for each biological replicate, of relative MATR3 and YBX1 protein levels normalized to loading control from autophagy inhibited MEFs, assayed by immunoblotting.

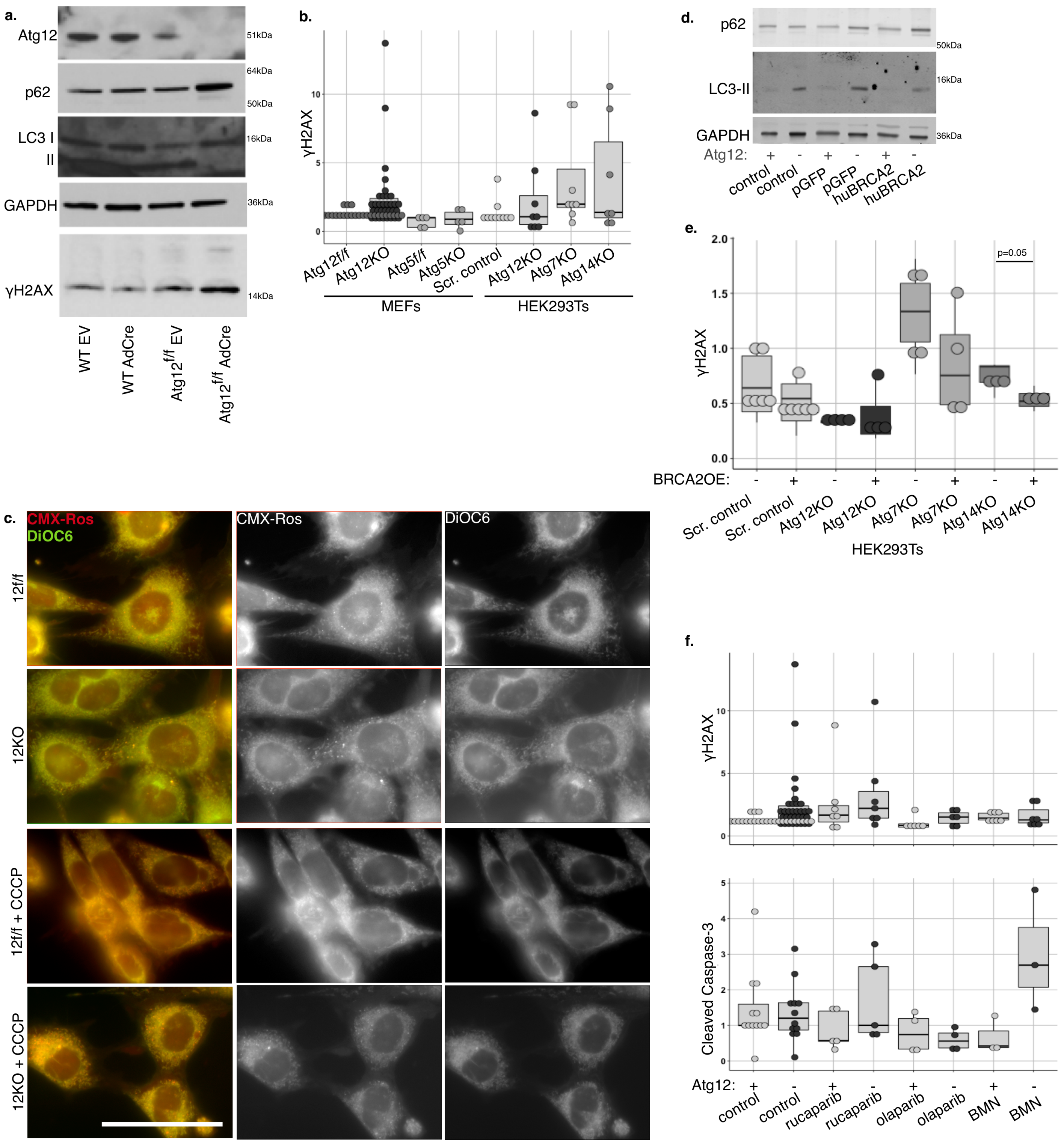

Supplementary Figure 8: Autophagy inhibition causes increased DNA damage independent of ROS, and sensitizes cells to PARP inhibitors (related to Figure 8)

a. WT MEFs and Atg12$^{f/f}$ MEFs were infected with Adenovirus expressing empty vector or Cre and 5d post infection protein lysate was collected and immunoblotted for ATG12, markers of autophagic flux (p62/SQSTM1, LC3), and DNA damage (γH2AX) as indicated.

b. Protein lysate was collected from MEFs and HEK293Ts that were genetically deleted for essential autophagy genes. Relative γH2AX protein levels as detected by immunoblot, normalized to loading control, were quantified and boxplot with dotplot overlay for each biological replicate is shown. There was no statistical difference ($p>0.05$) by ANOVA with Tukey's HSD post hoc test.

c. $Atg12^{f/f}$ or $Atg12^{KO}$ MEFs were stained for mitochondria using MitoTracker Red CMX-Ros (500nM for 15min), and membrane potential of mitochondria by DiOC6(3) (10ng/ml for 5min), under normal conditions and following CCCP treatment (50μM for 30min). Representative immunofluorescence image is shown, three independent biological experiments were performed. Bar = 50μm.

d. Protein lysate was collected from MEFs with ectopic expression of either GFP or human BRCA2, and immunoblotted for markers of autophagy.

e. Ectopic expression of either GFP (BRCA2OE negative) or human BRCA2 (BRCA2OE positive) in HEK293T cells lacking the indicated Atgs was performed. Relative γH2AX protein levels, normalized to loading control, as detected by immunoblotting were quantified and a boxplot with dotplot overlay for each biological replicate is shown. Statistical significance was assessed by t test between GFP and BRCA2 overexpression.

f. $Atg12^{f/f}$ or $Atg12^{KO}$ MEFs were treated for 16h with vehicle control, or the PARP inhibitors rucaparib (100nM), olaparib (100nM) or BMN (2nM) prior to lysis. Boxplot with dotplot overlay for each biological replicate is shown for relative γH2AX and cleaved caspase 3 protein levels, normalized to loading control, as detected by immunoblotting. There was no statistical difference ($p>0.05$) by ANOVA with Tukey's HSD post hoc test.

Supplementary Table 1: Pearson correlation values between biological replicates for RP

		12f/f Control	12f/f HBSS	12KO control	12KO HBSS
**Pearson of RNA read counts**	rep1v2	0.999493374	0.999655533	0.999749177	0.999889177
	rep1v3	0.999933962	0.99993667	0.999790718	0.999974191
	rep1v4	0.999797444	0.99976291	0.999794633	0.999676187
	rep2v3	0.999581019	0.999677894	0.999886499	0.999912464
	rep2v4	0.999850359	0.999296039	0.999916345	0.999759433
	rep3v4	0.999774091	0.999795222	0.999783269	0.999652037
**Pearson of RP read counts**	rep1v2	0.989011405	0.997650192	0.984537039	0.995001589
	rep1v3	0.999164542	0.999787873	0.996697571	0.999473952
	rep1v4	0.998593311	0.999556942	0.993837845	0.999463327
	rep2v3	0.99058821	0.997904474	0.993283945	0.996755611
	rep2v4	0.993829078	0.998741683	0.994877077	0.996547
	rep3v4	0.998606925	0.999573337	0.998666655	0.999548399
**Pearson of P-val from within replicates**	rep1v2	0.91123548	0.677373189	0.751806731	0.754952357
	rep1v3	0.869337775	0.801813334	0.827749197	0.787217358
	rep1v4	0.879017764	0.766726411	0.810161346	0.795316853
	rep2v3	0.844229194	0.585342583	0.797549924	0.759961983
	rep2v4	0.882464613	0.706773882	0.831379563	0.819737239
	rep3v4	0.860238439	0.757845313	0.889925915	0.790899669

Uncropped gels

Figure 1A

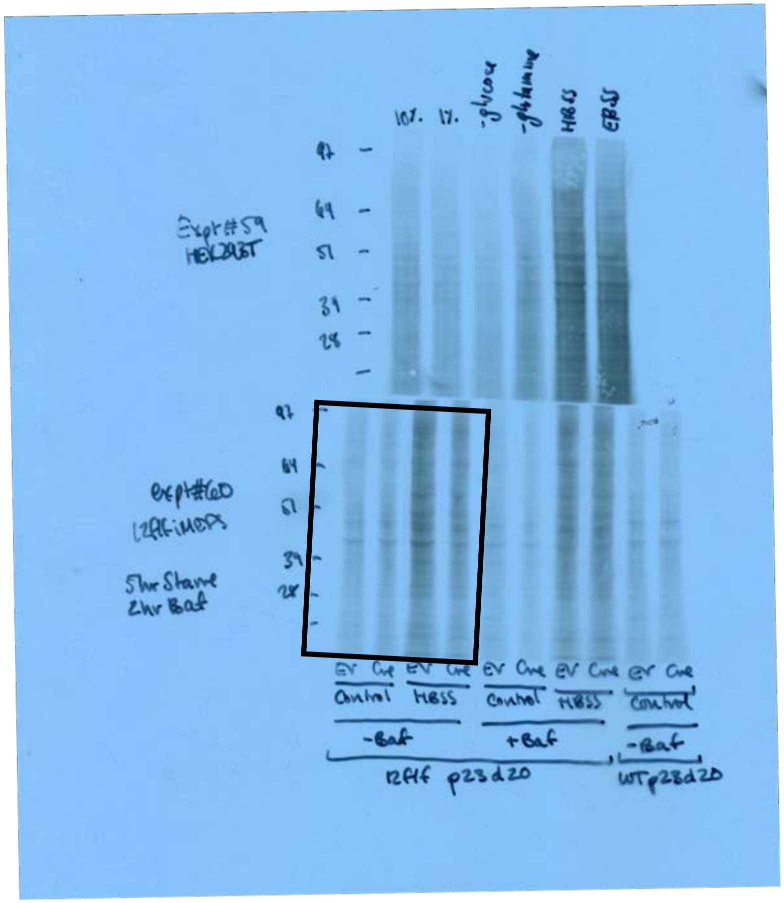

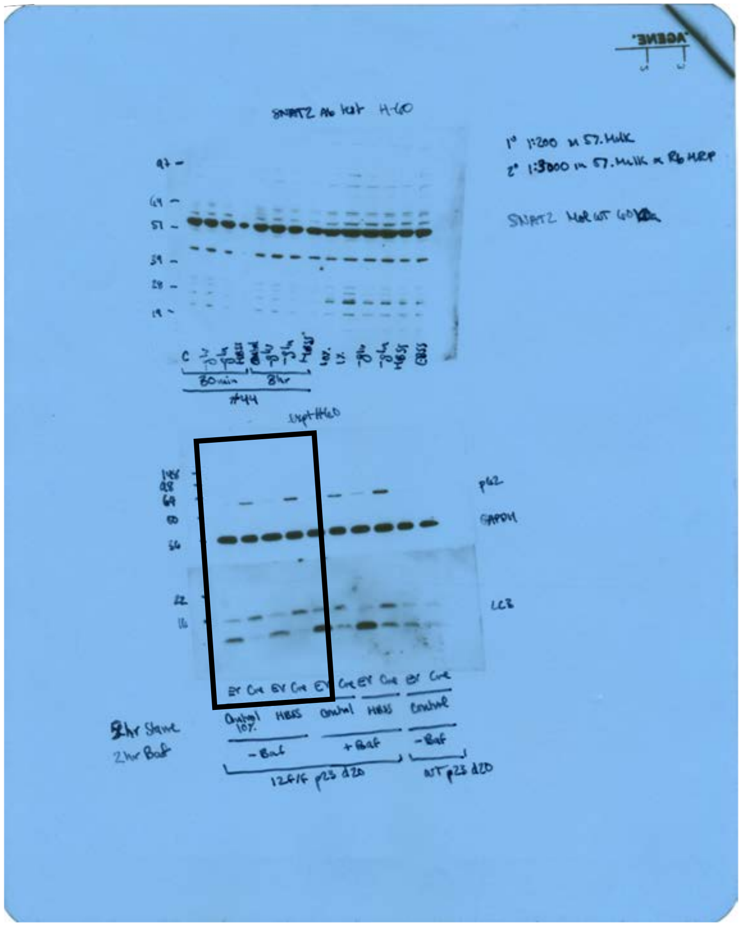

Figure 1C

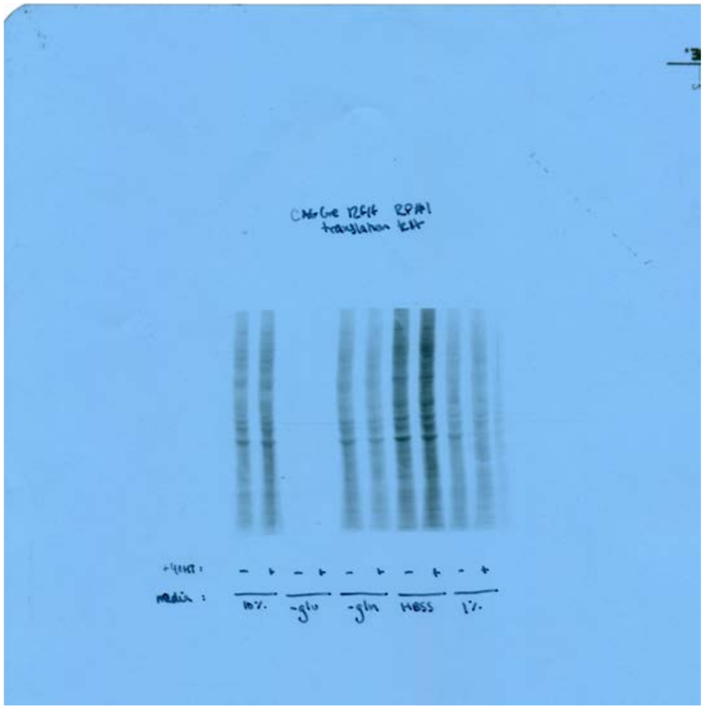

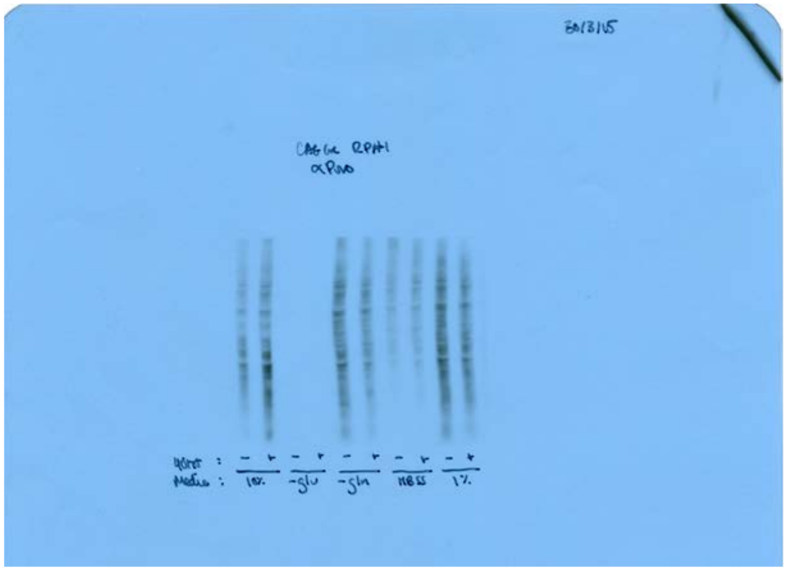

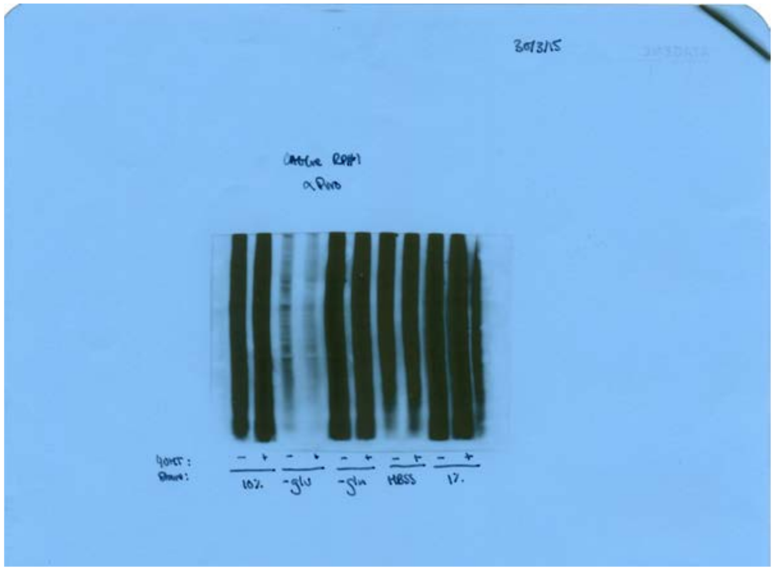

Figure 1E

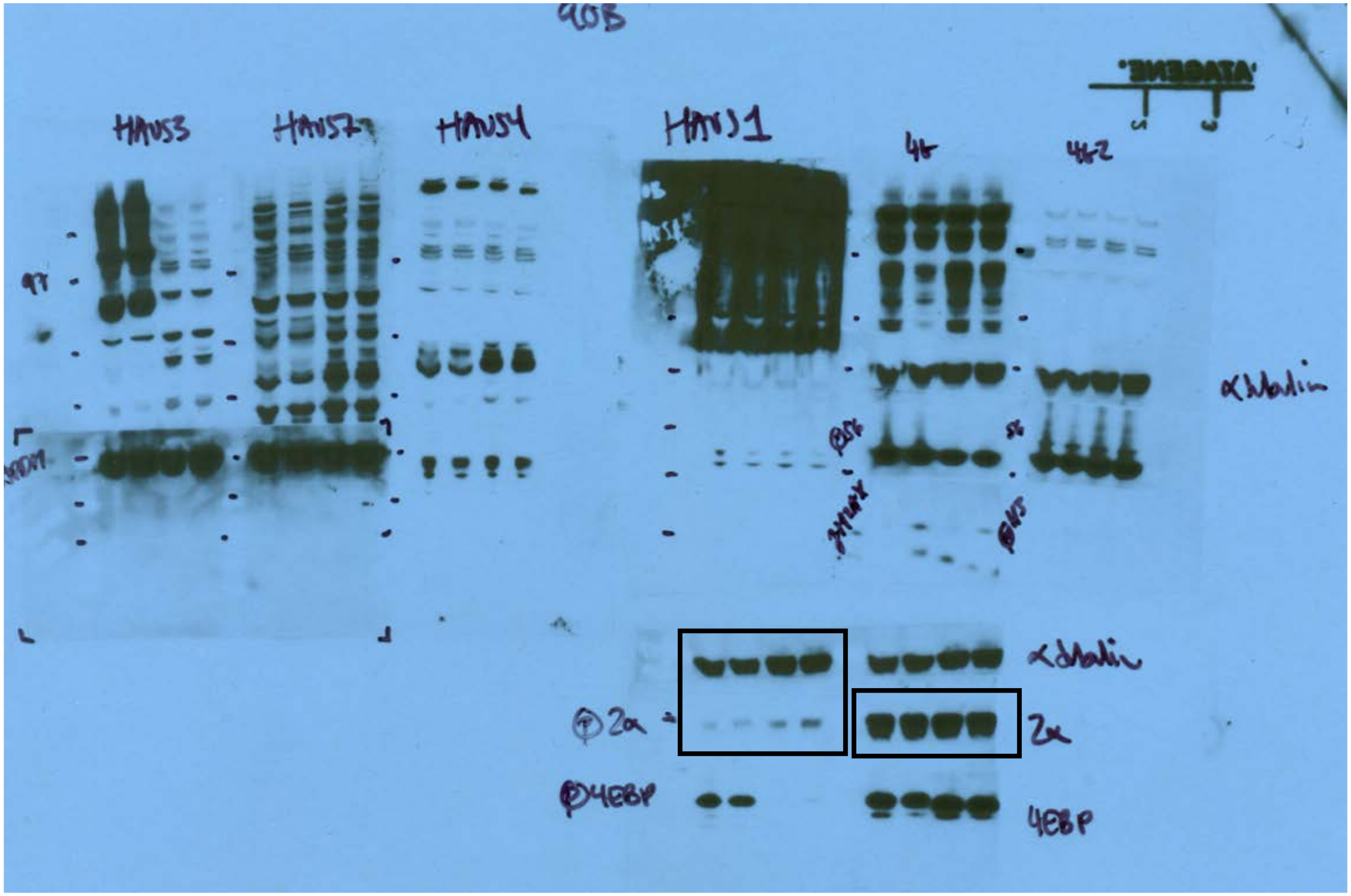

Figure 1H

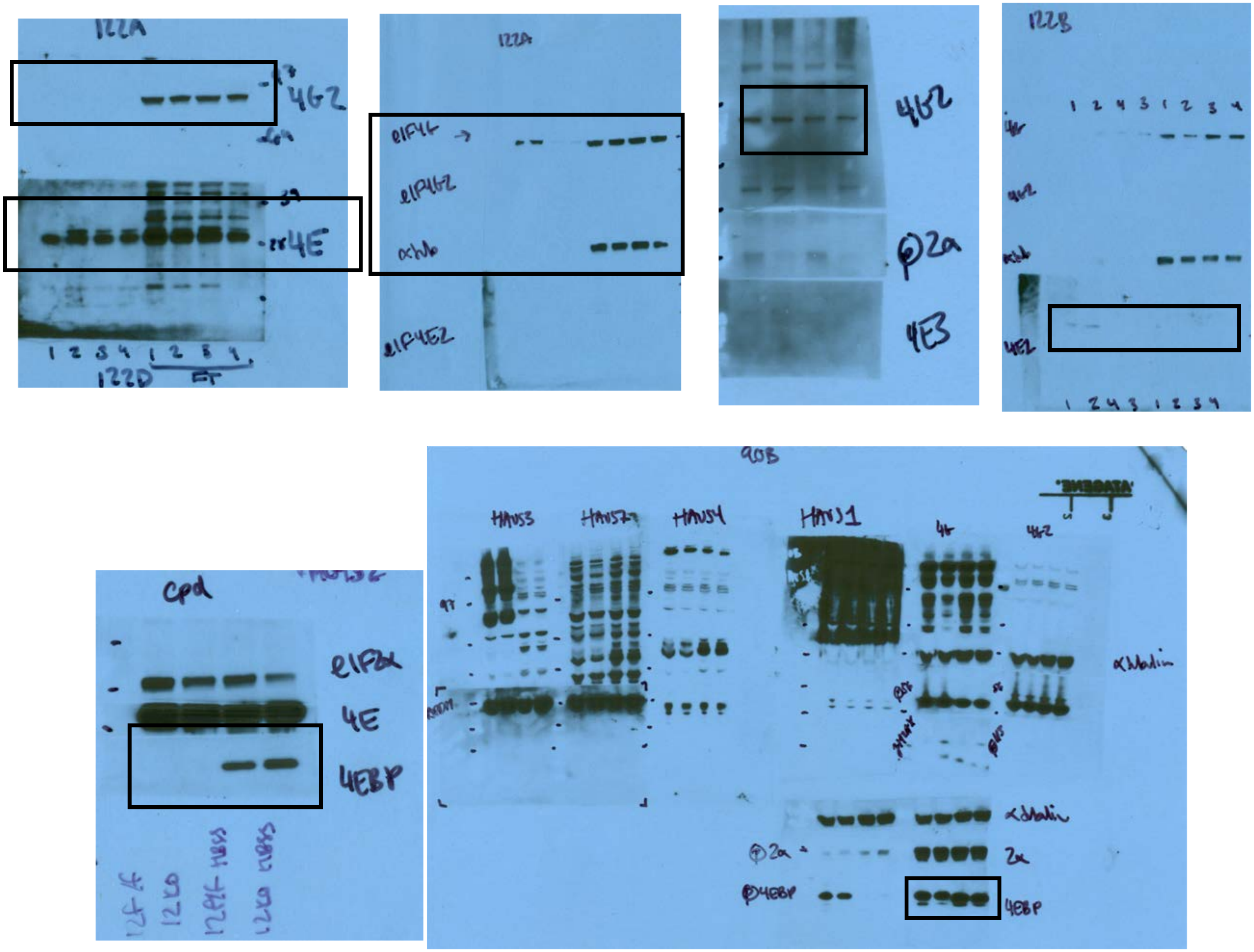

Figure 2B

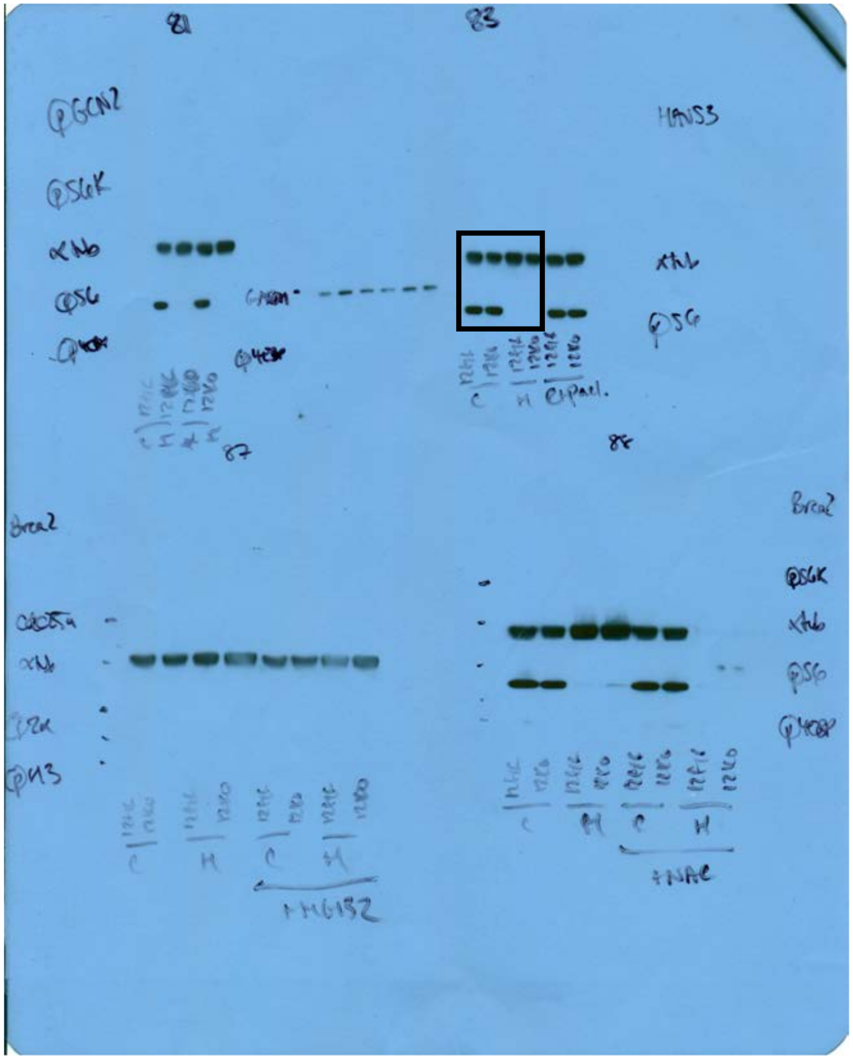

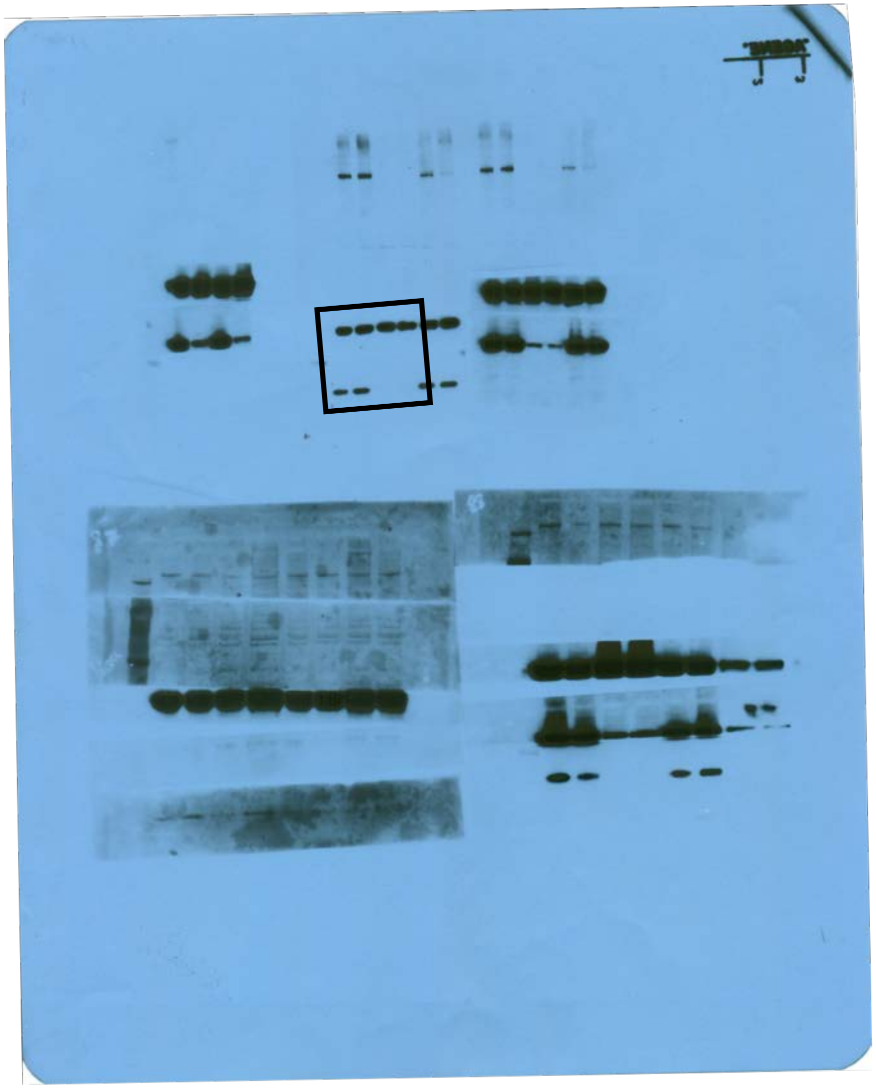

Figure 2E

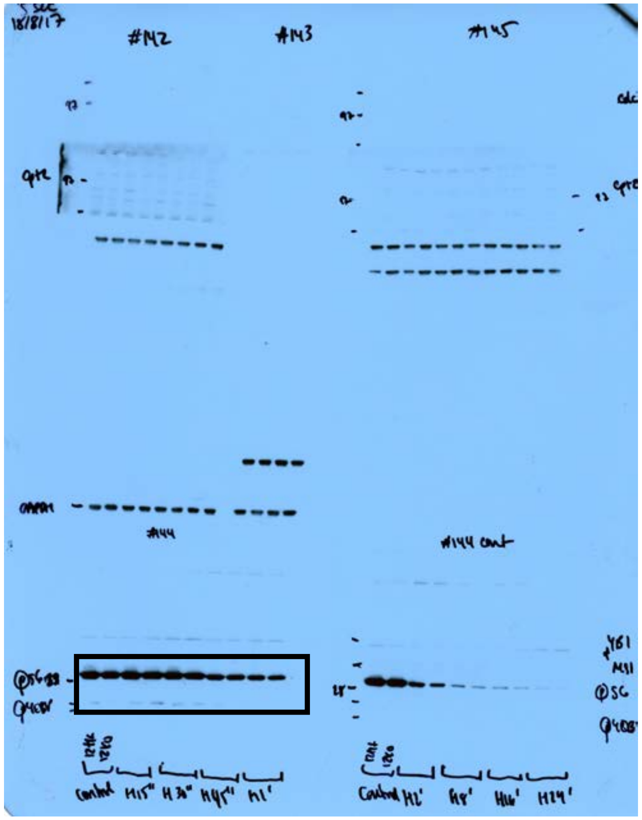

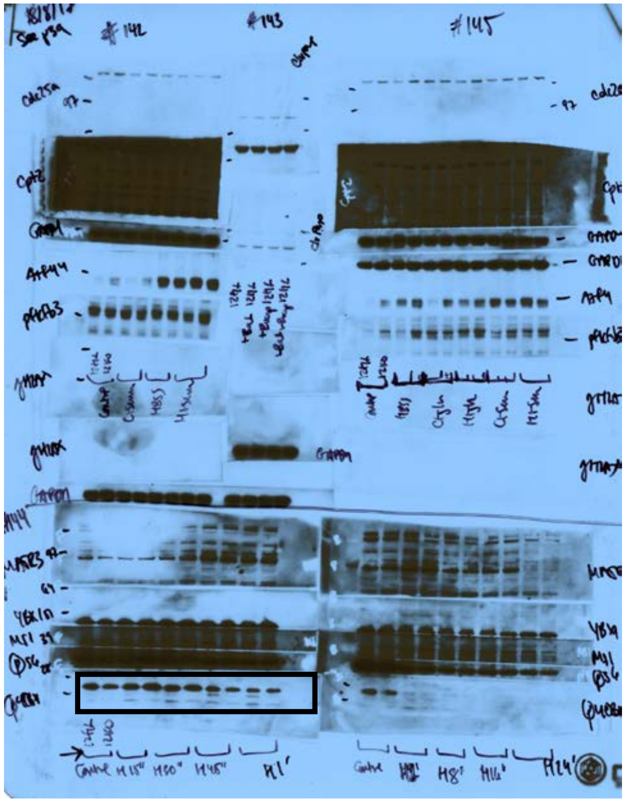

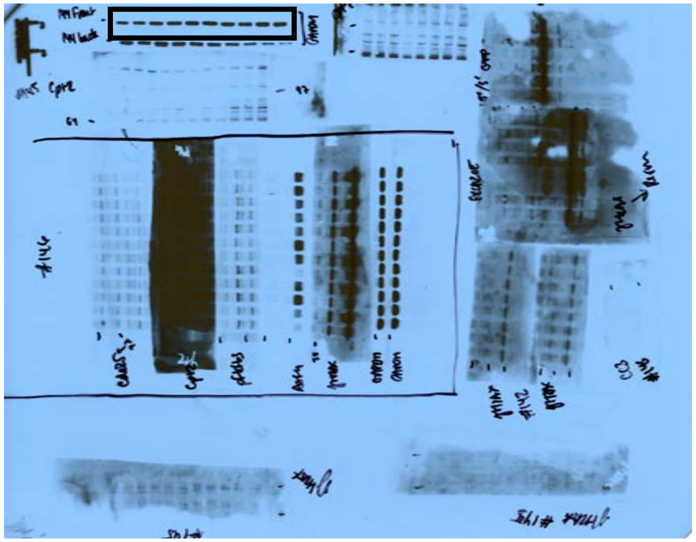

Figure 4A

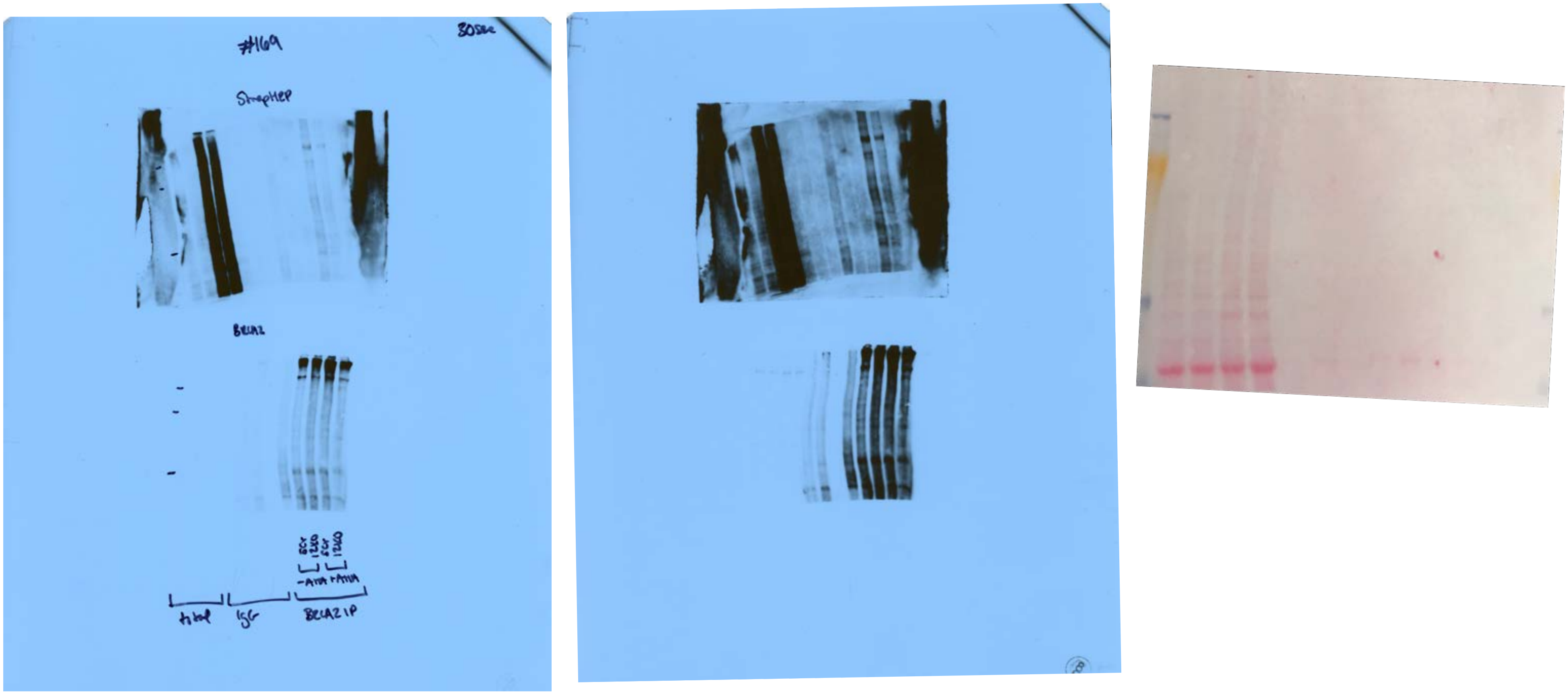

Figure 4C

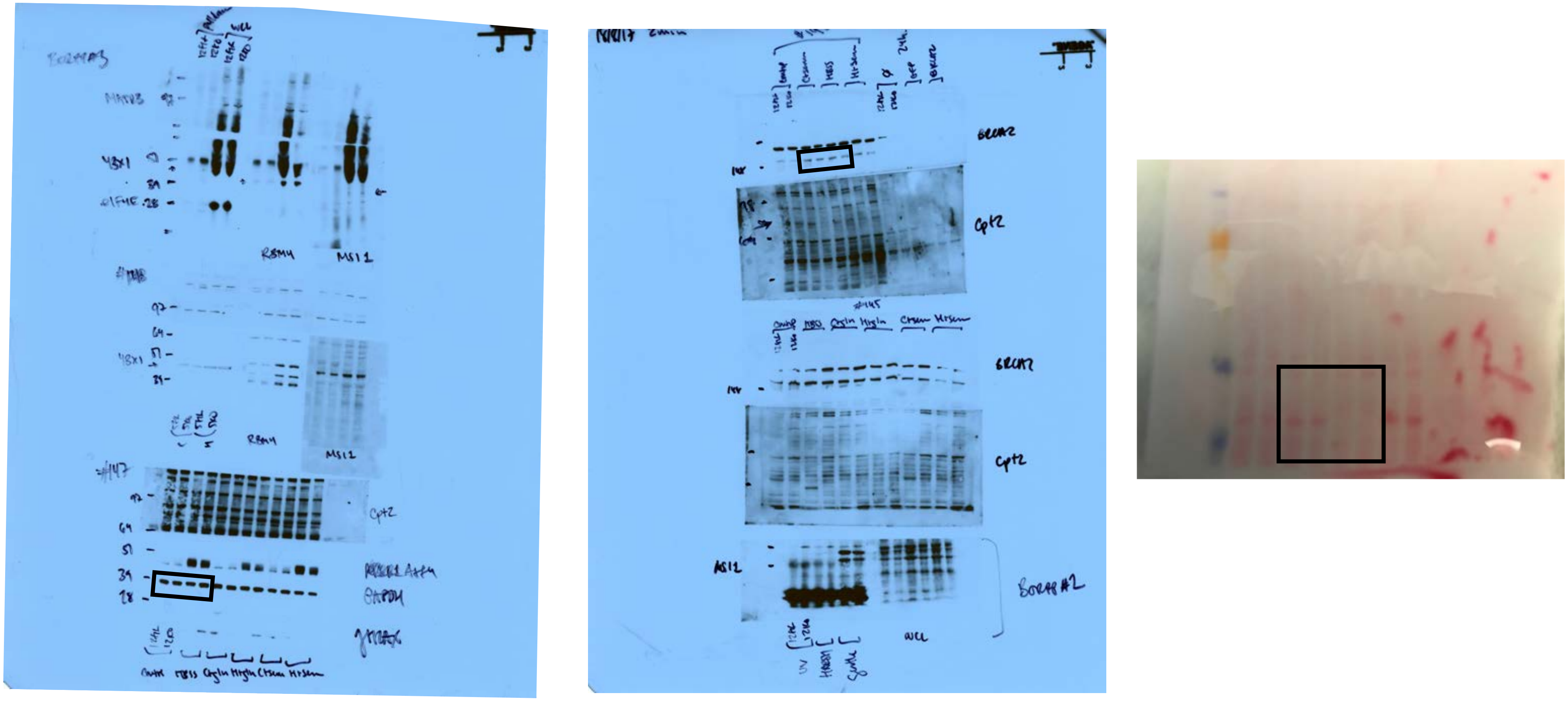

Figure 4E

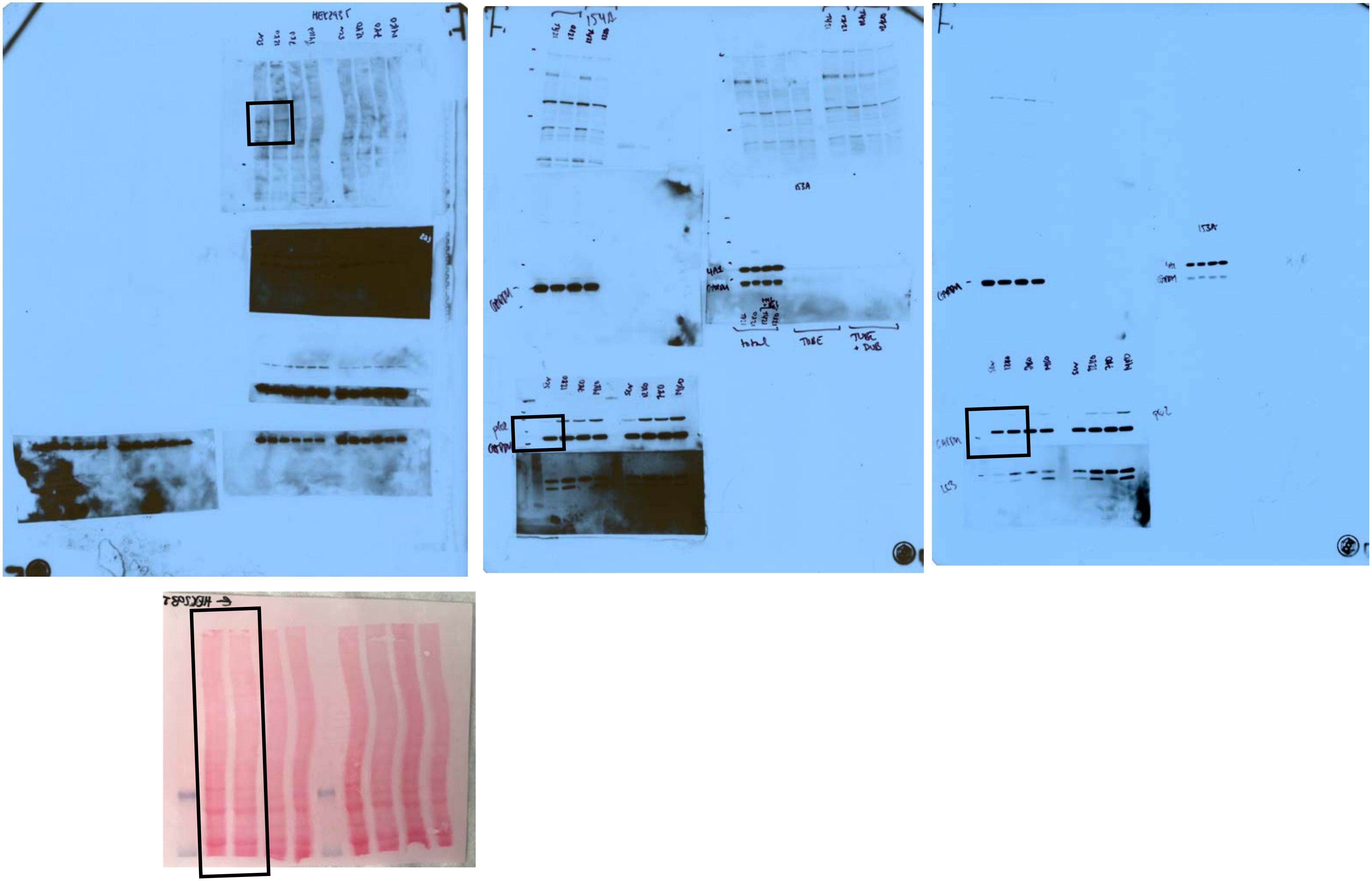

Figure 4H

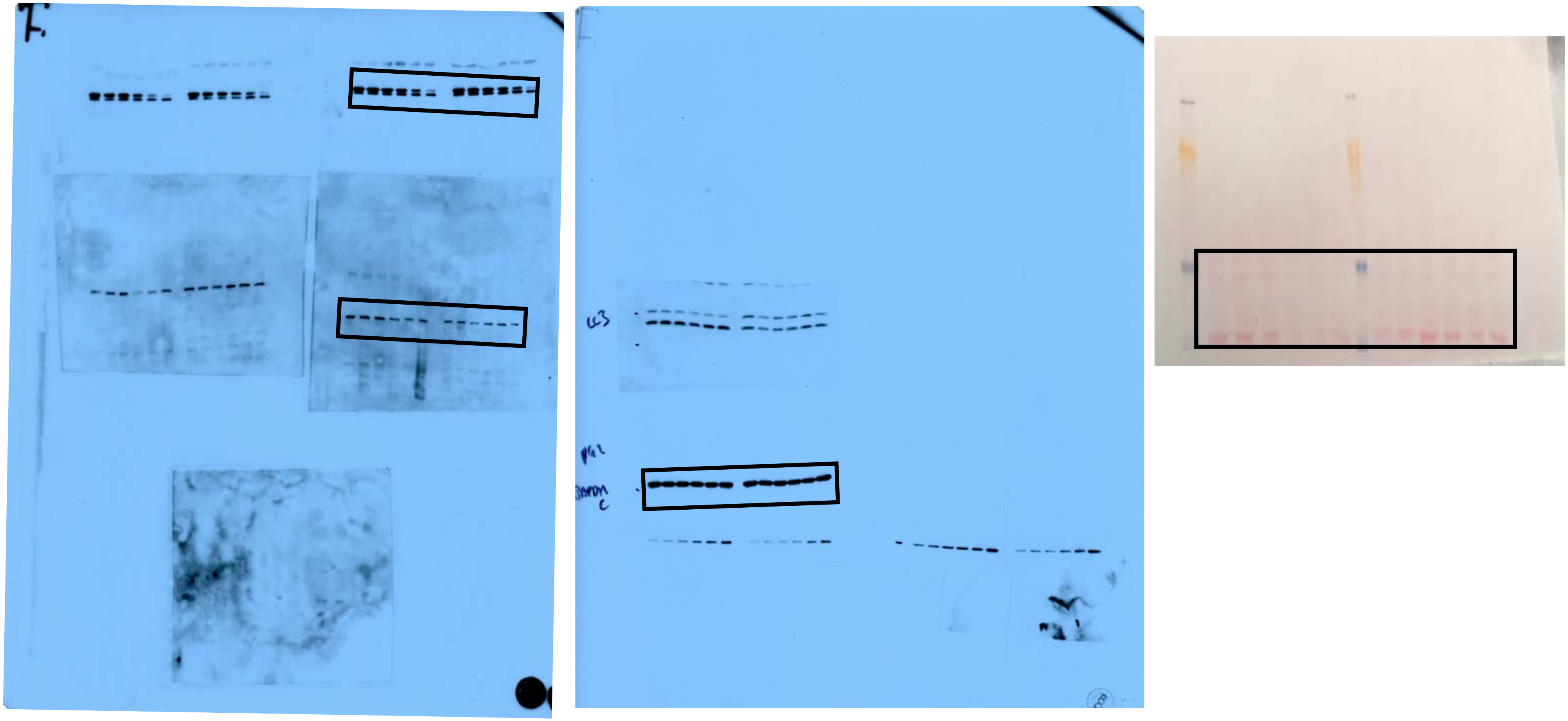

Figure 5A

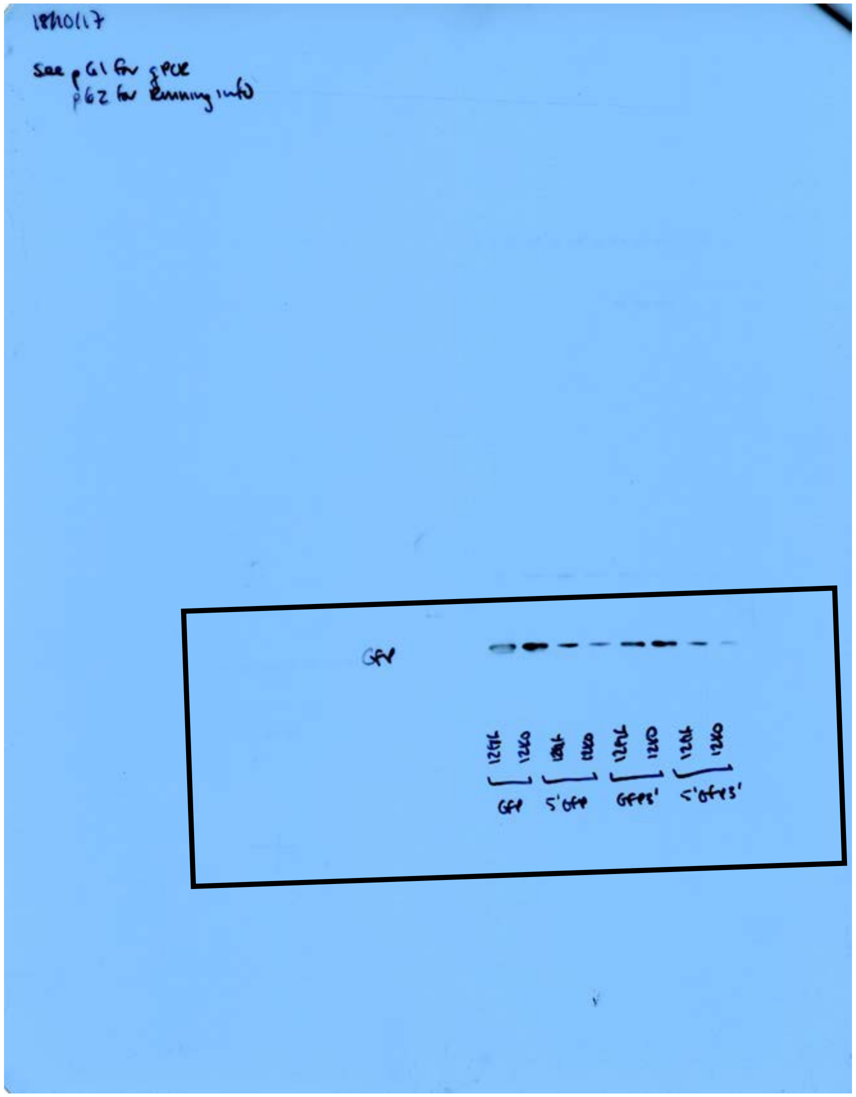

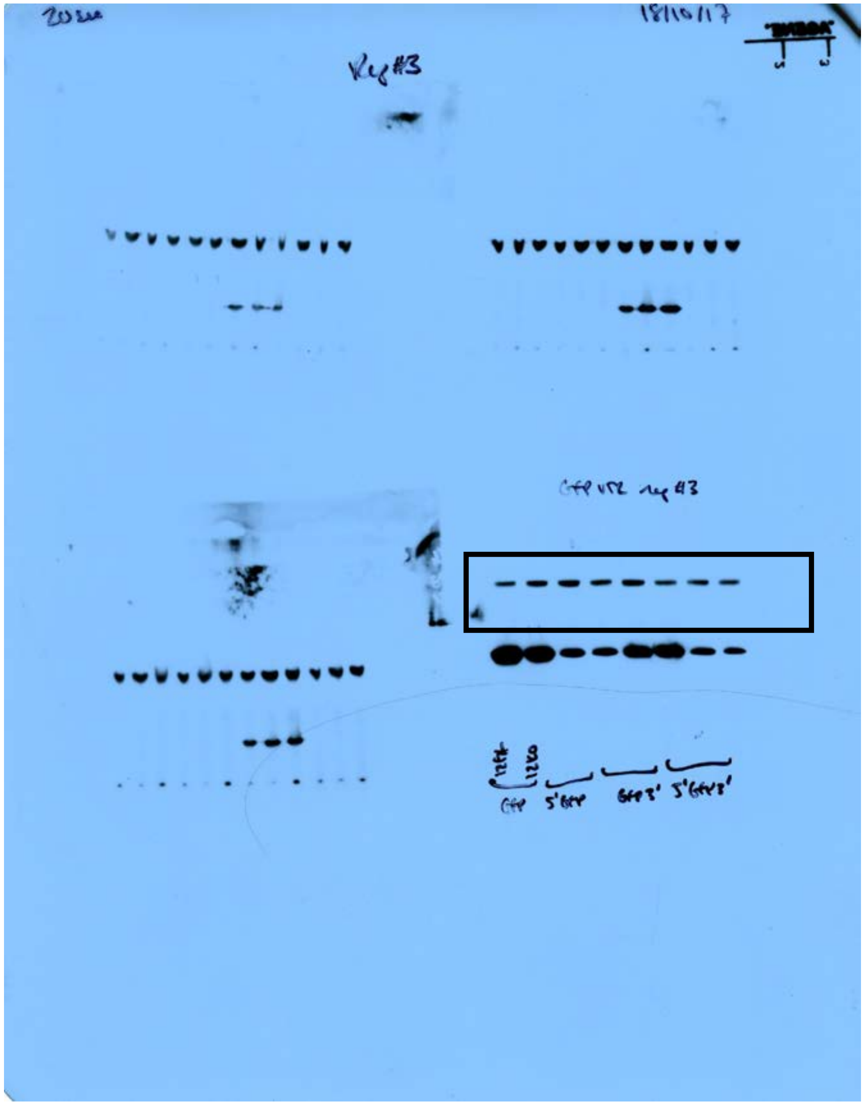

Figure 5E

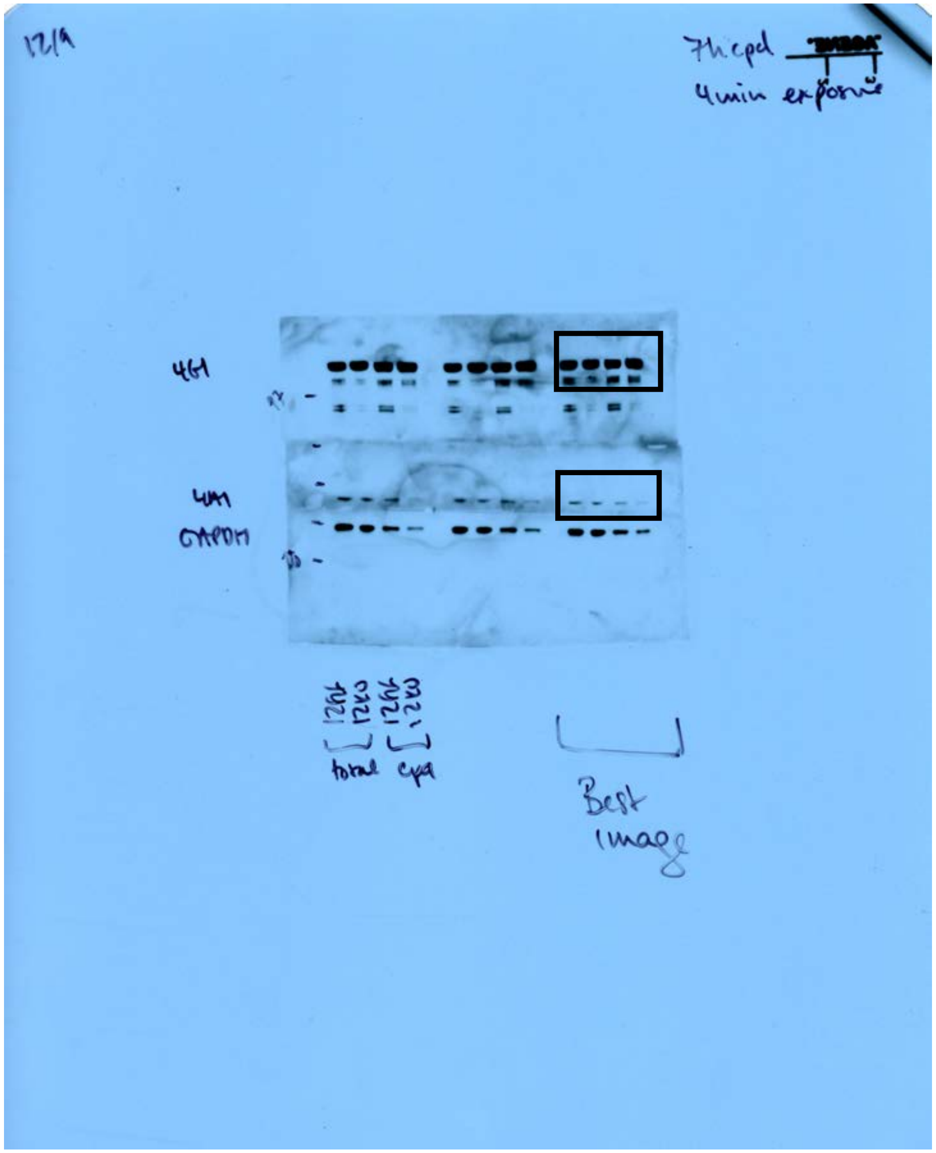

7hcpd
4min exposure
GAPDH
total
Best image

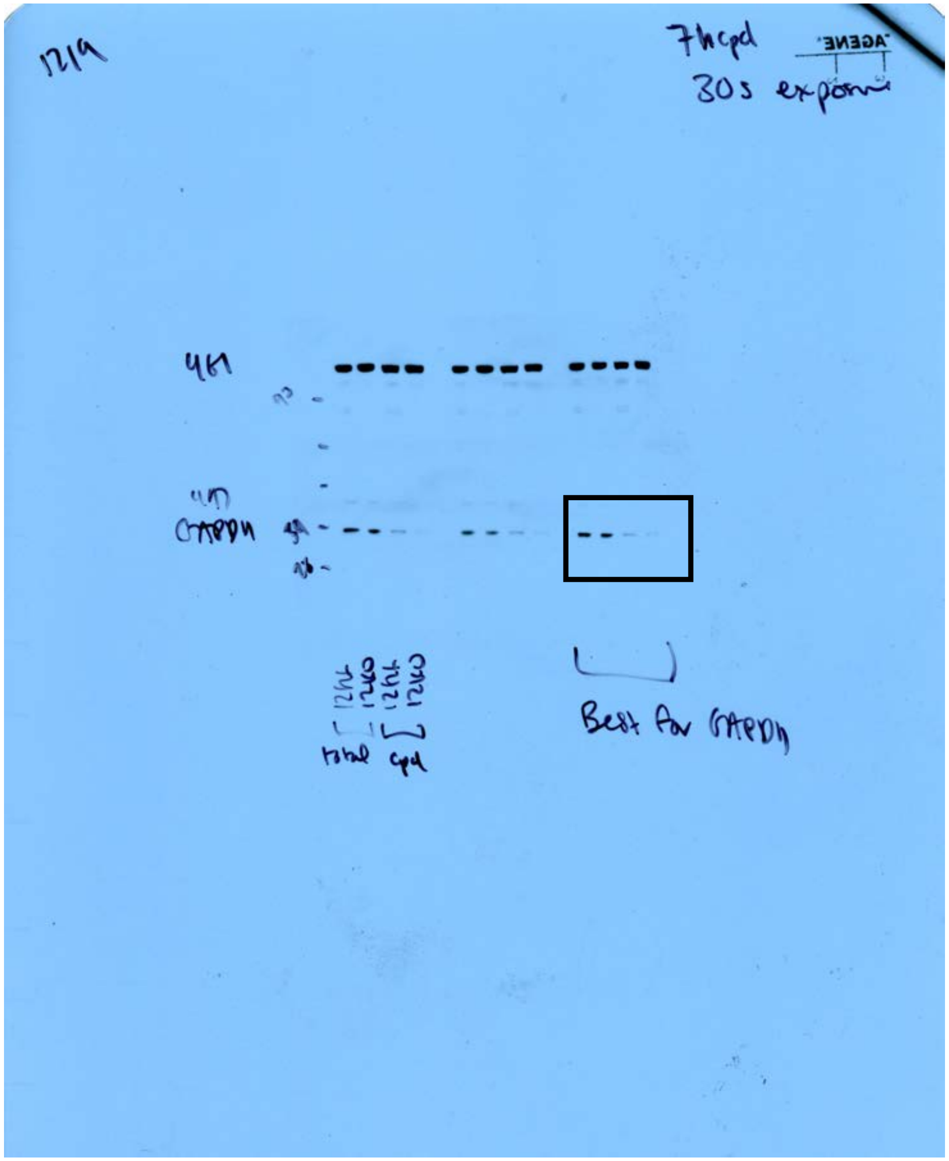

7hcpd
30s exposure
GAPDH
total
Best for GAPDH

Figure 6C

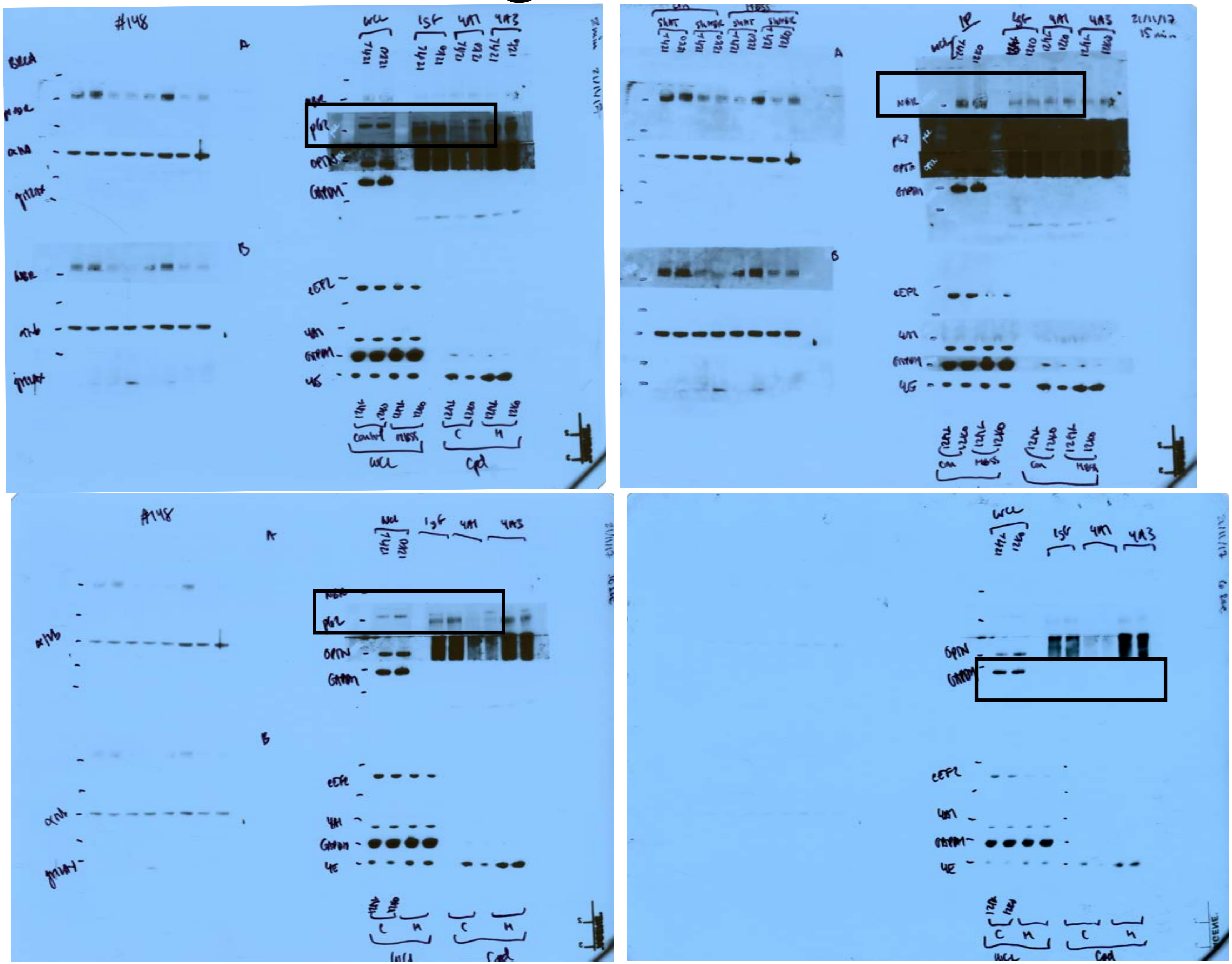

Figure 6E

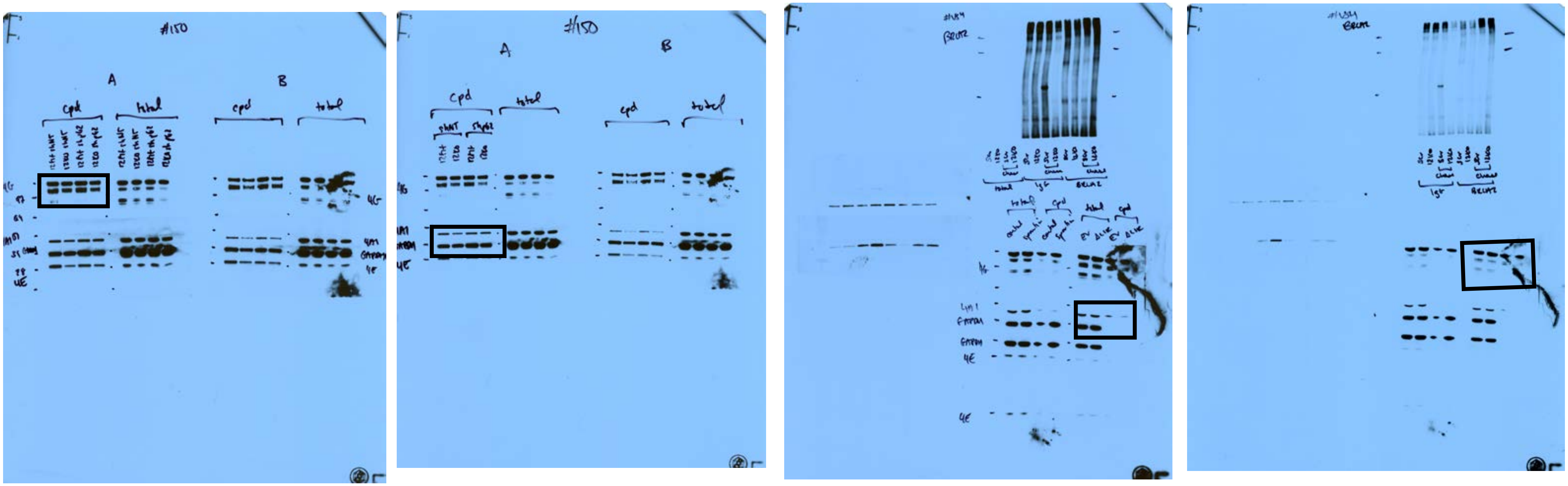

Figure 7B

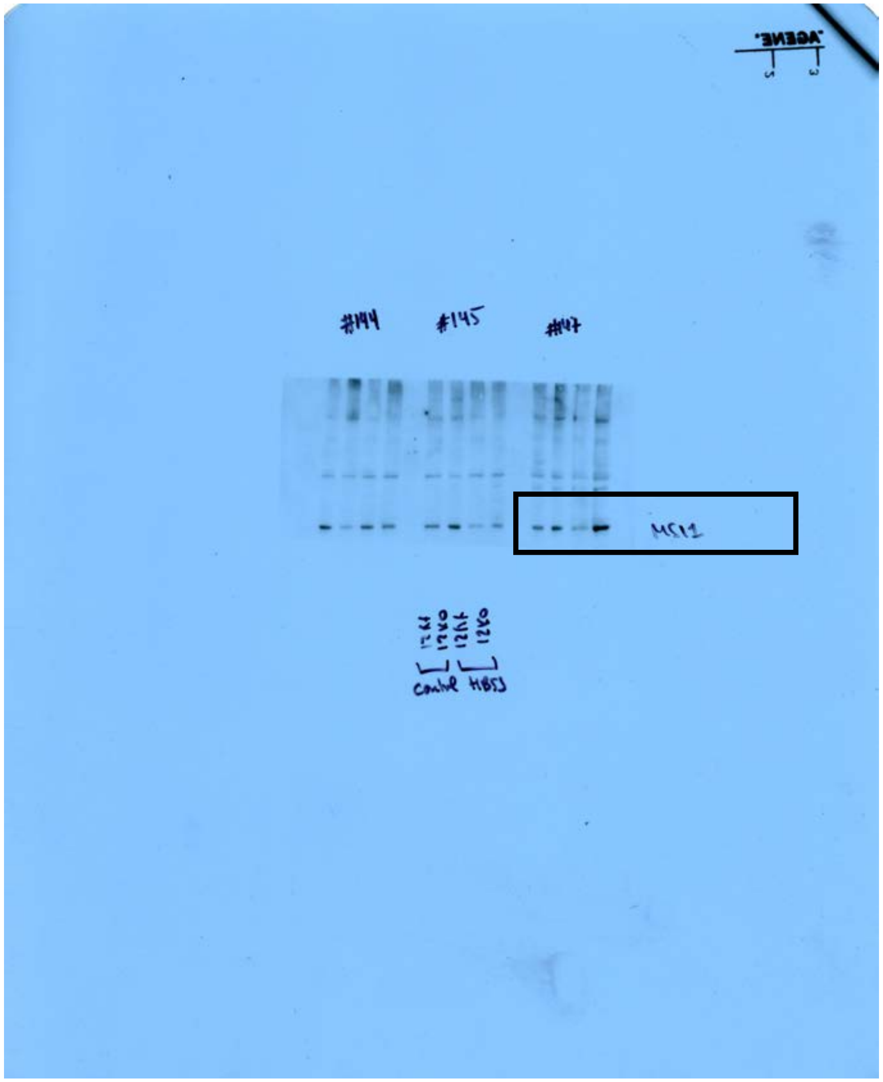

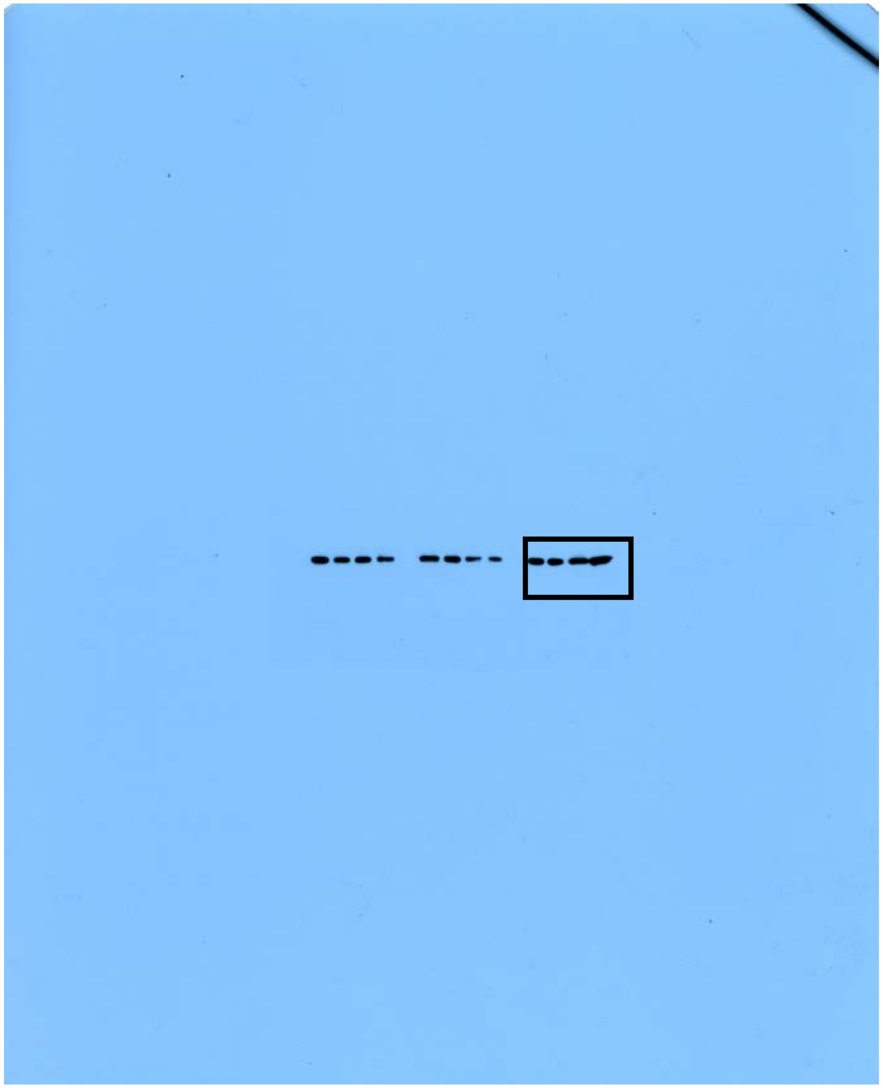

Figure 7C

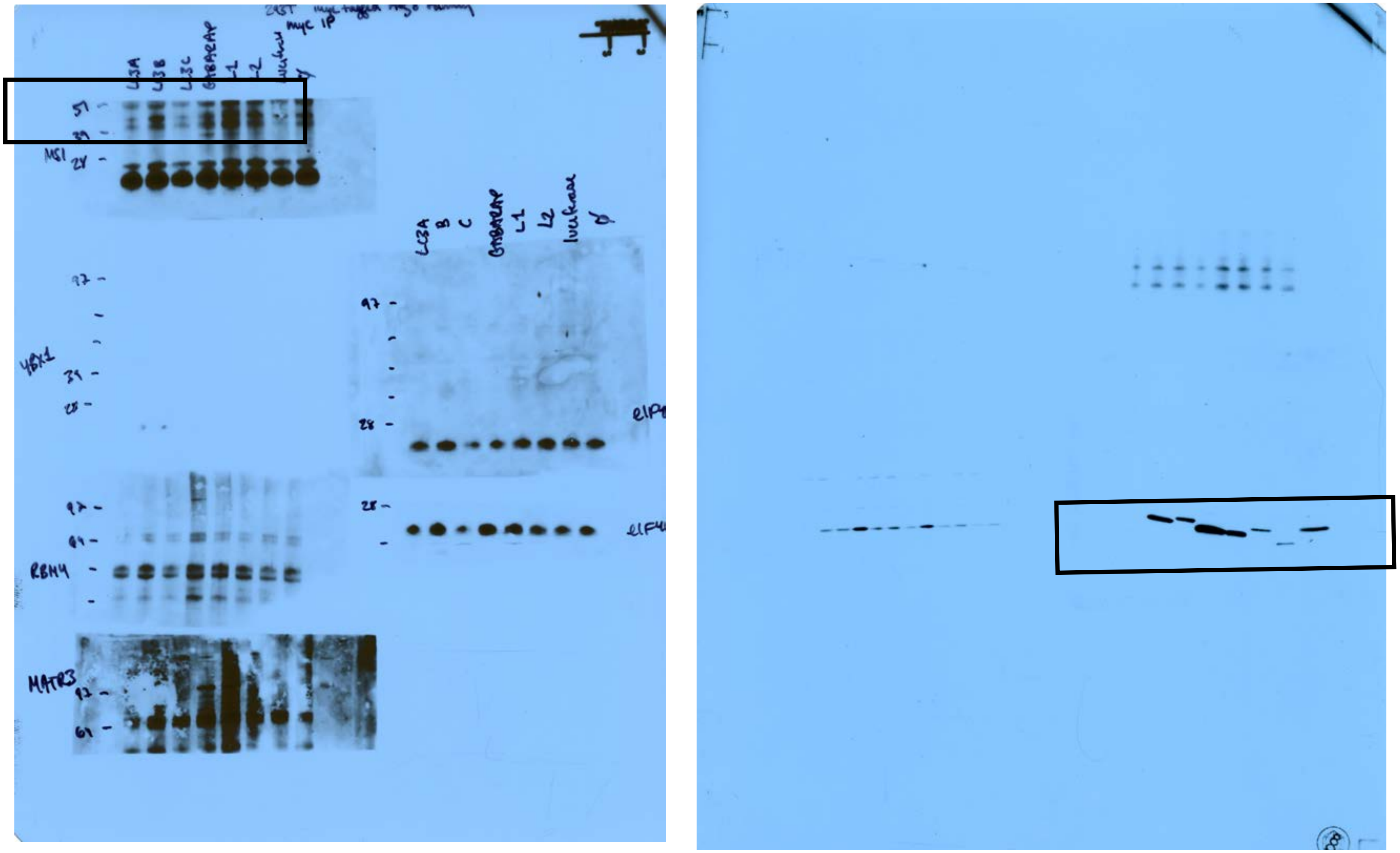

Figure 7D

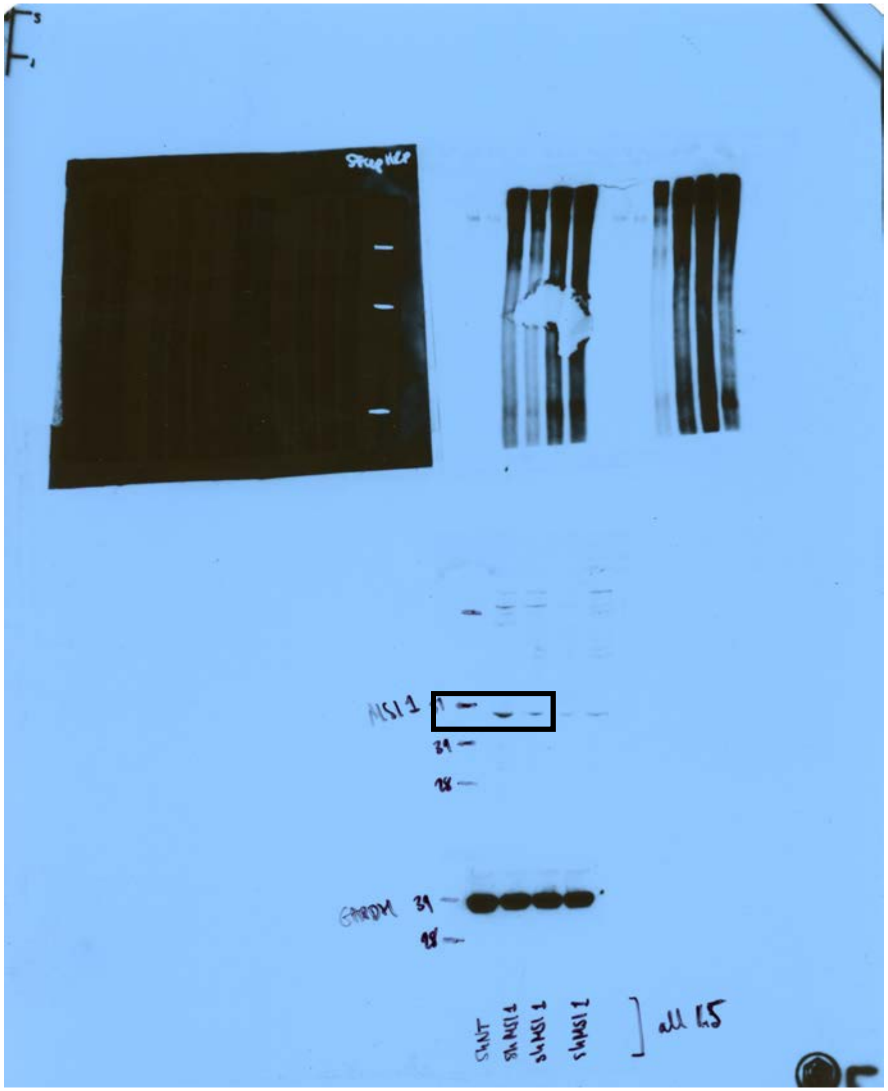
Strep HRP
MSI 1
39
28
GAPDH 39
28
all 1.5

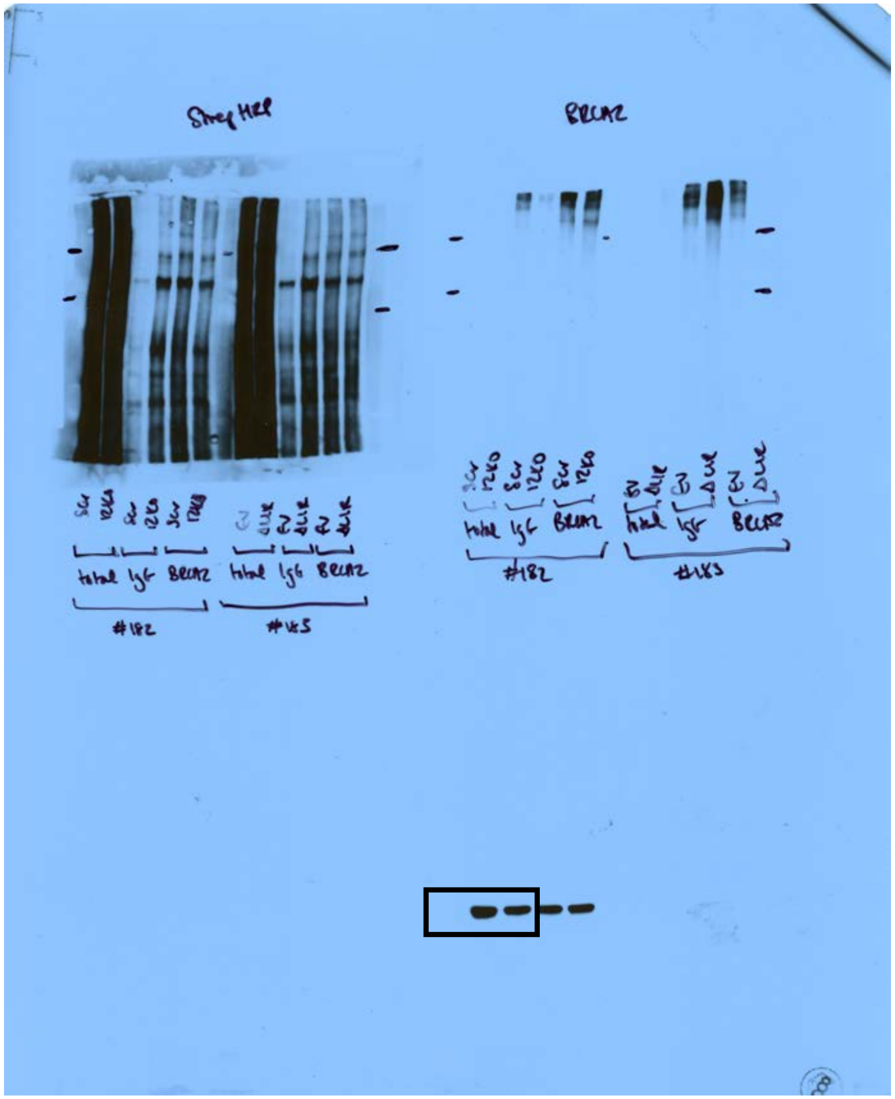
Strep HRP
BRCA2
total
IgG
BRCA2
#182
#183

Figure 7E

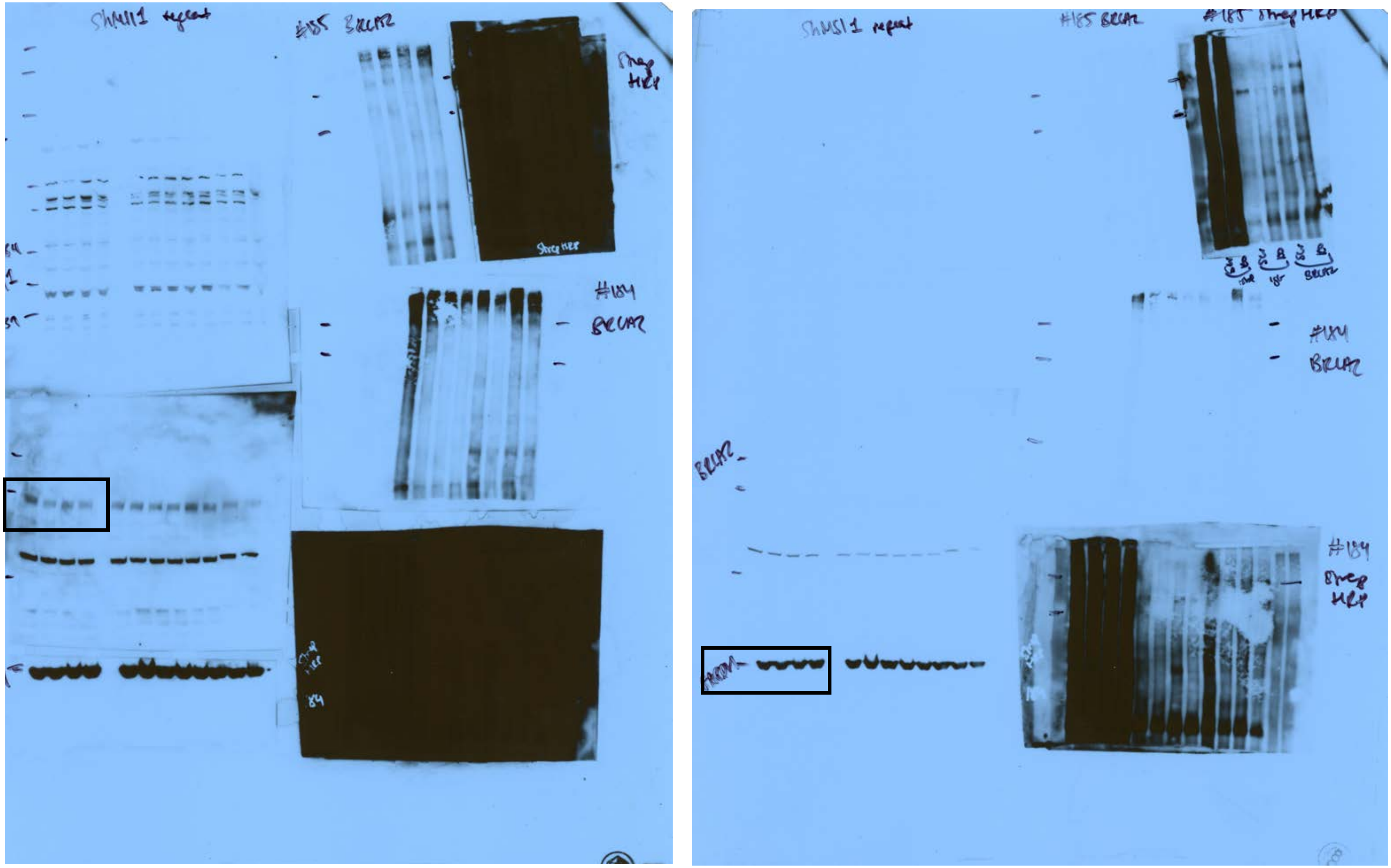

Figure 8D

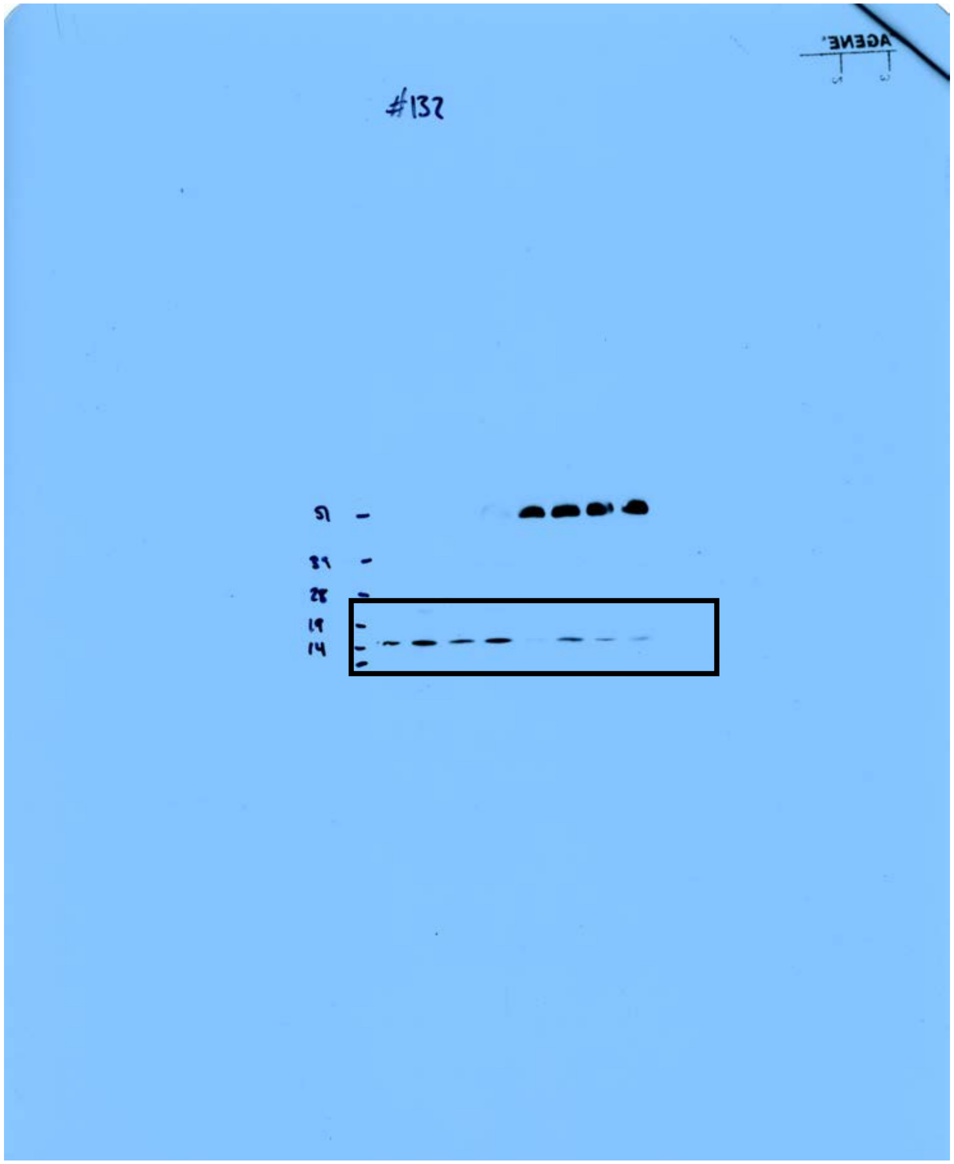

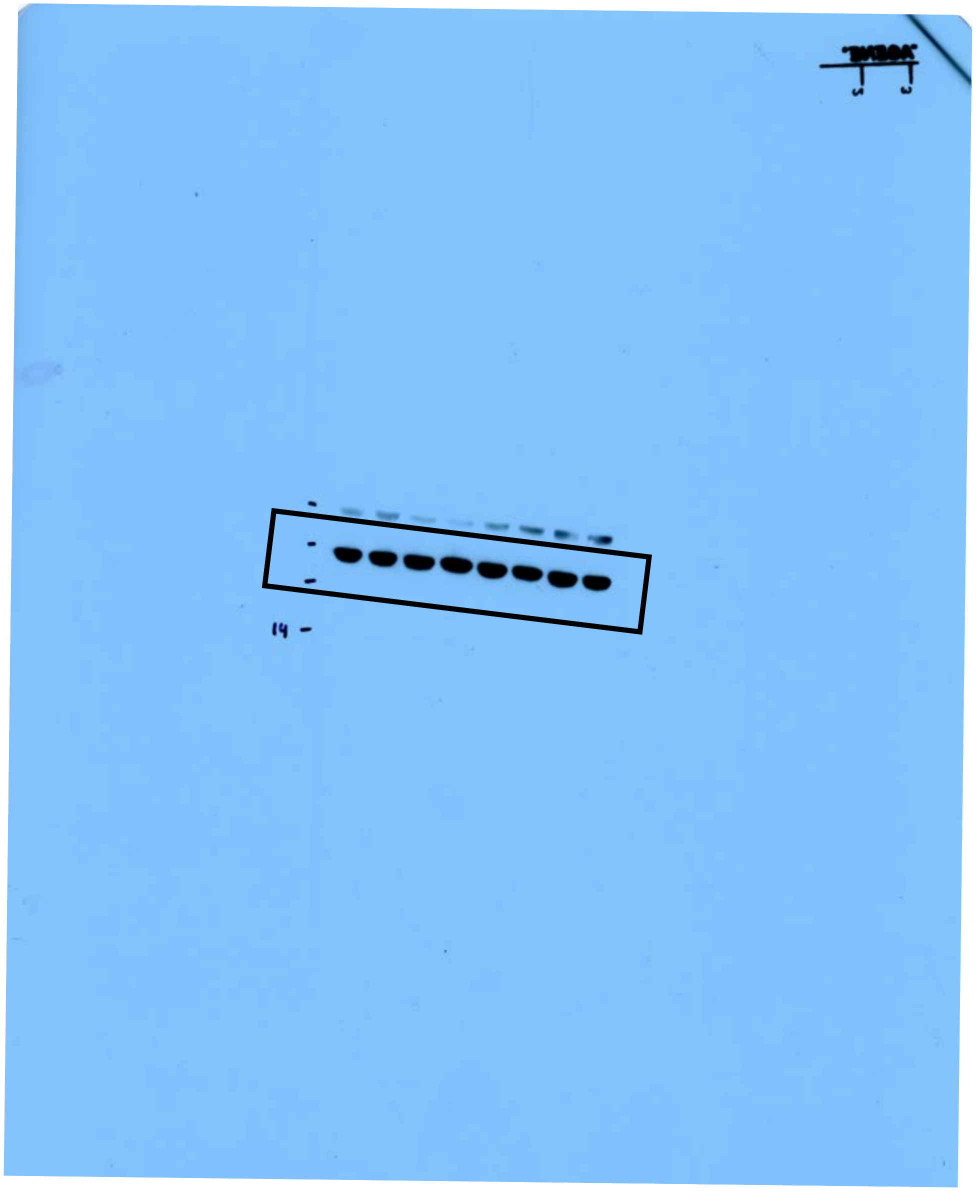

Figure 8E

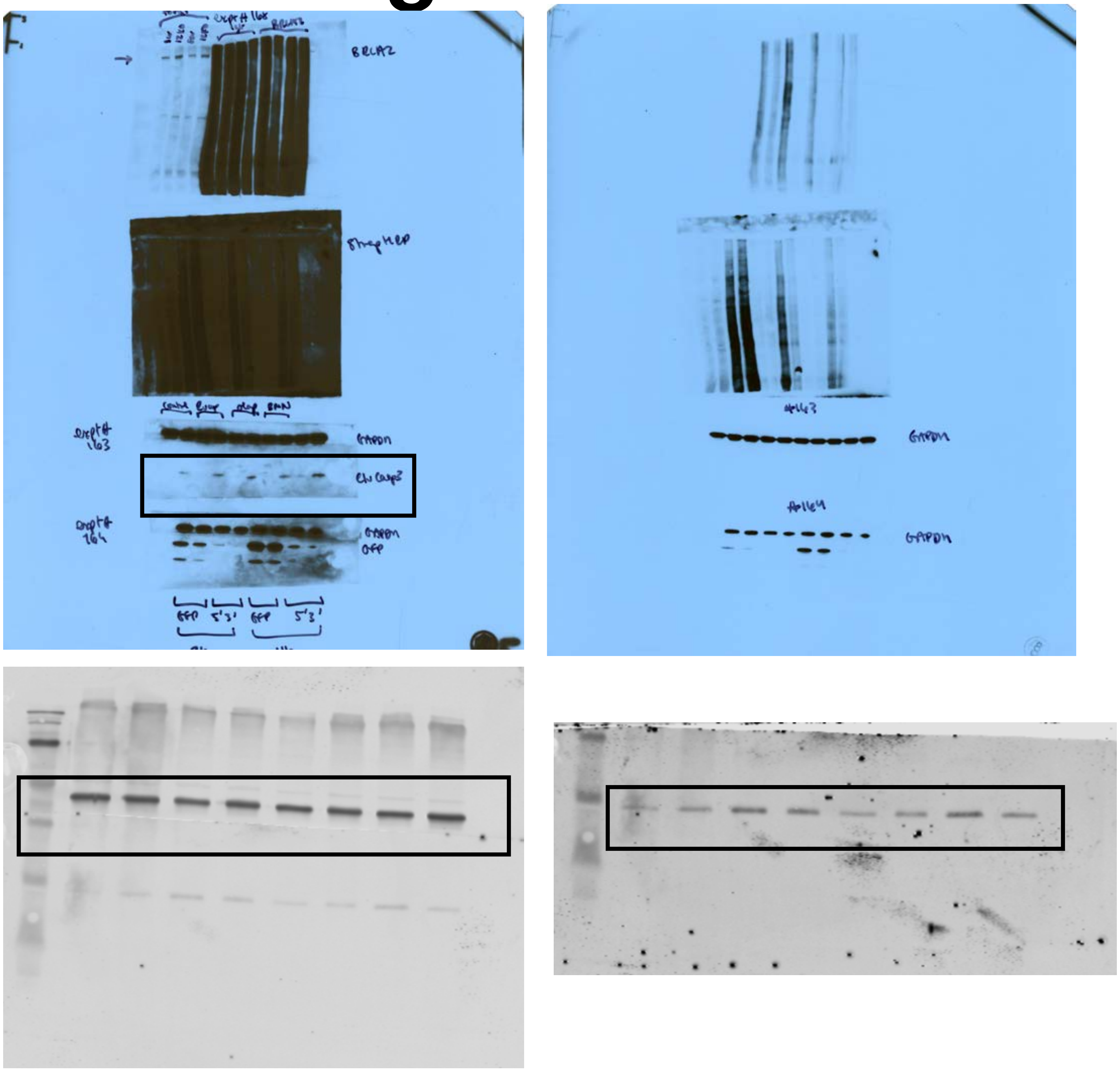

Figure 9B

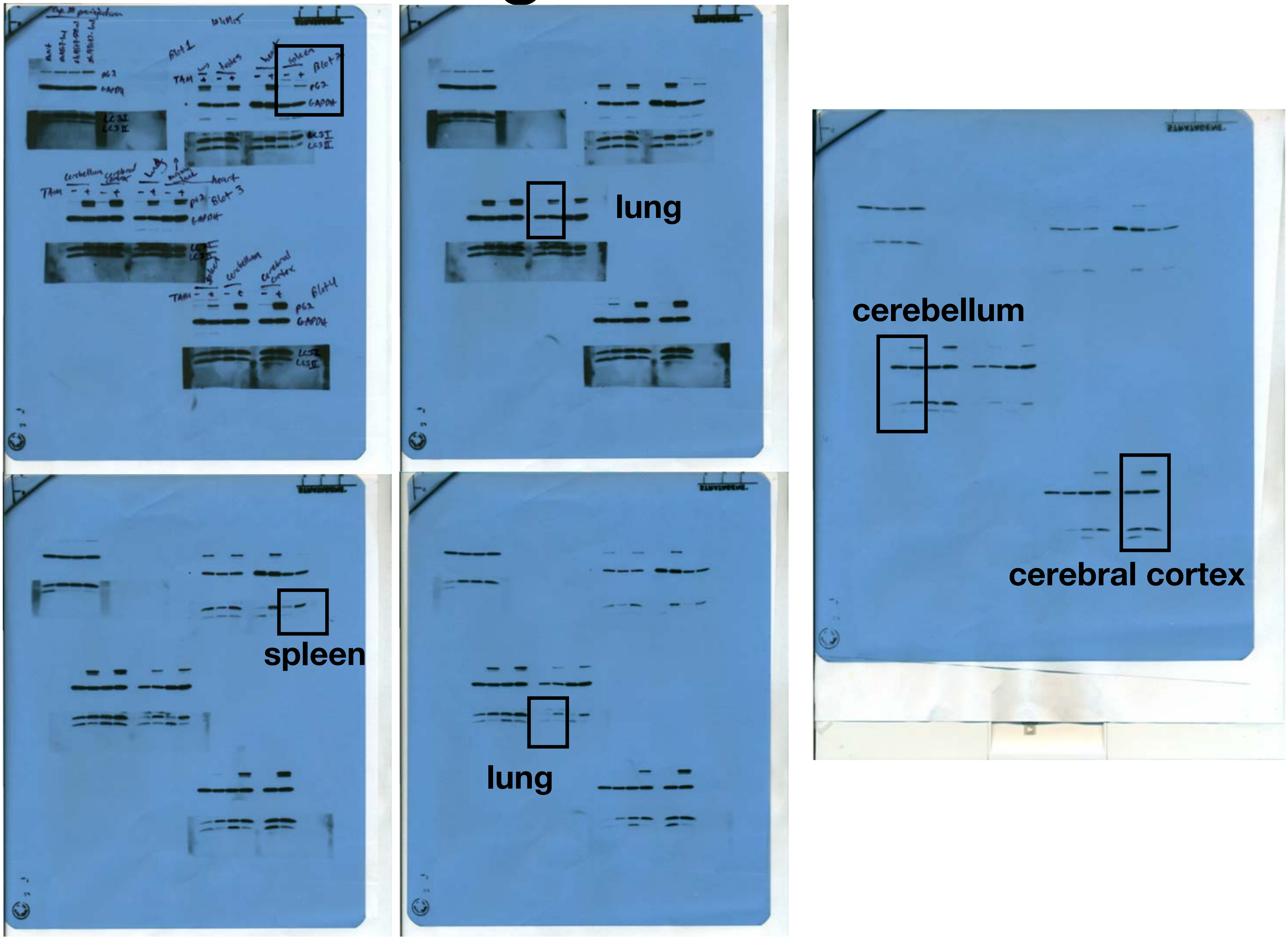

Figure 9B

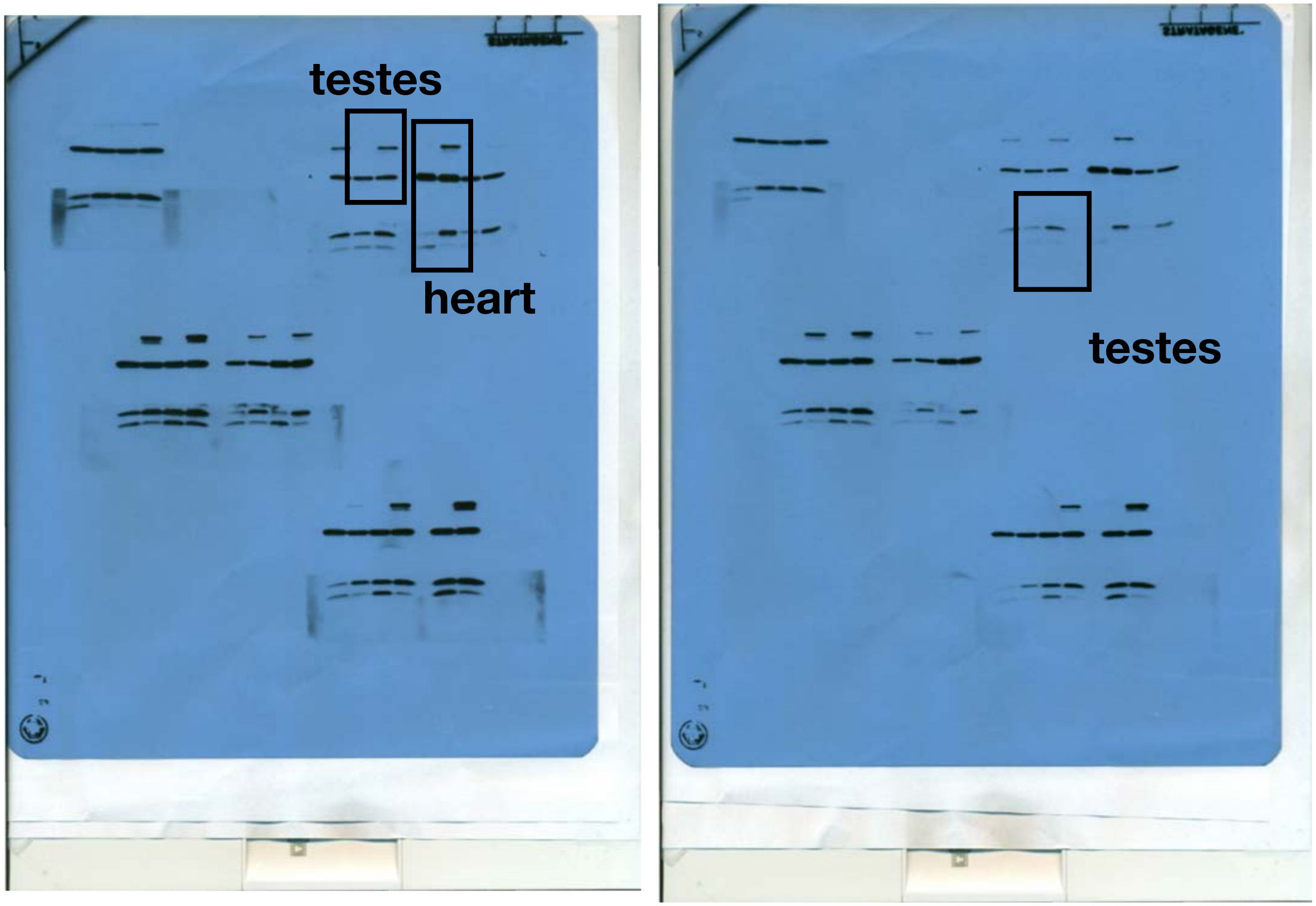

Figure 9B

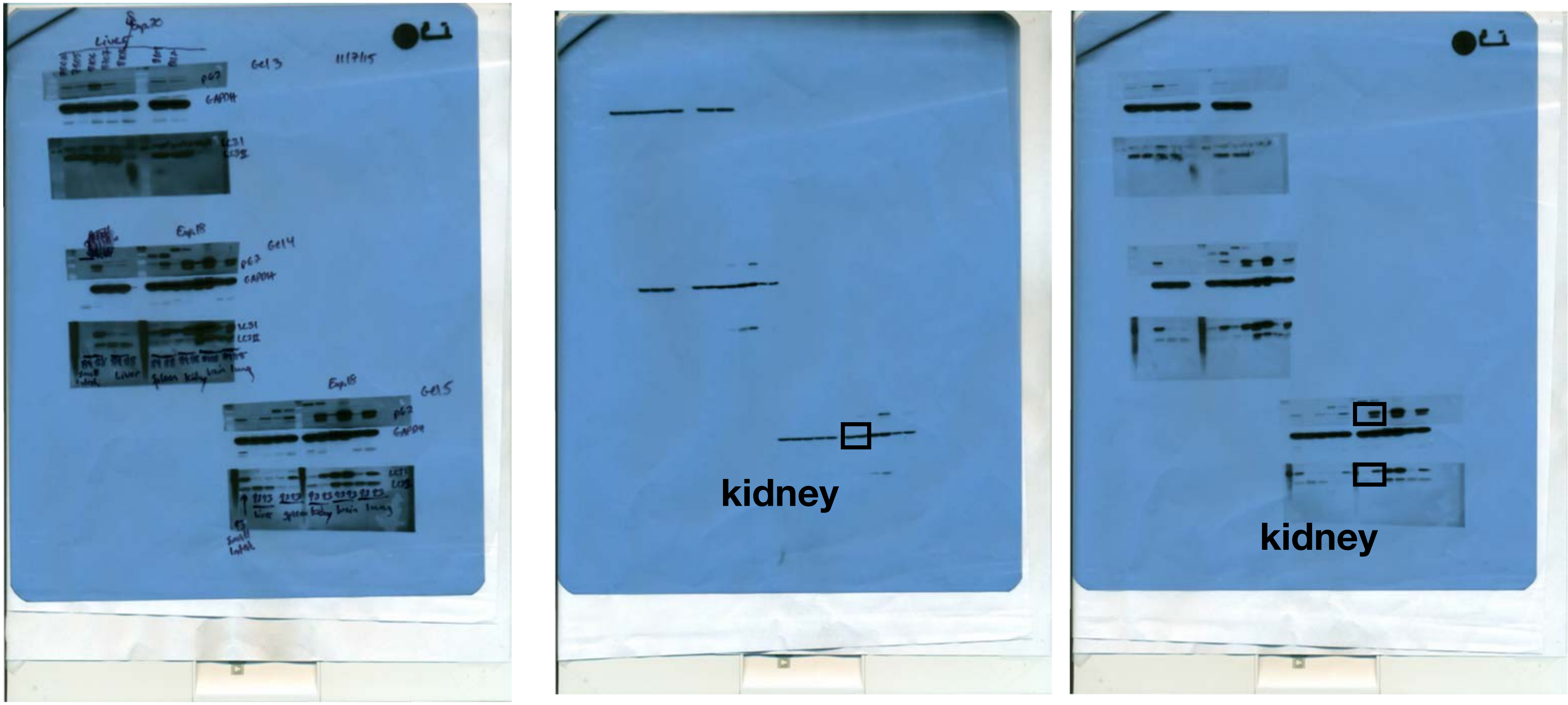
Liver
Gel 3
11/7/15
p62
GAPDH
Gel 4
p62
GAPDH
Gel 5
p62
GAPDH
kidney
kidney

Figure 9E

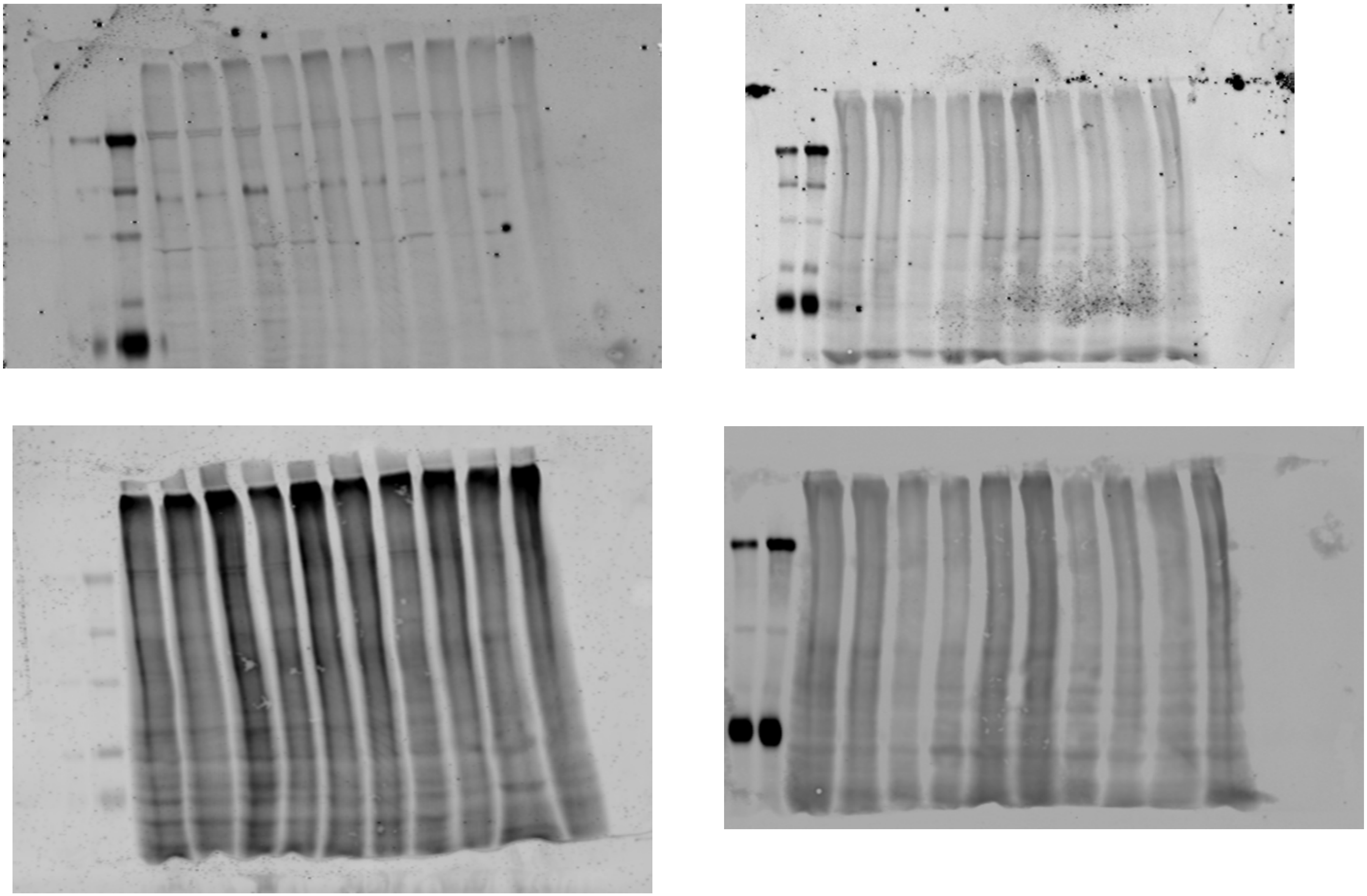

Figure S1 A,B

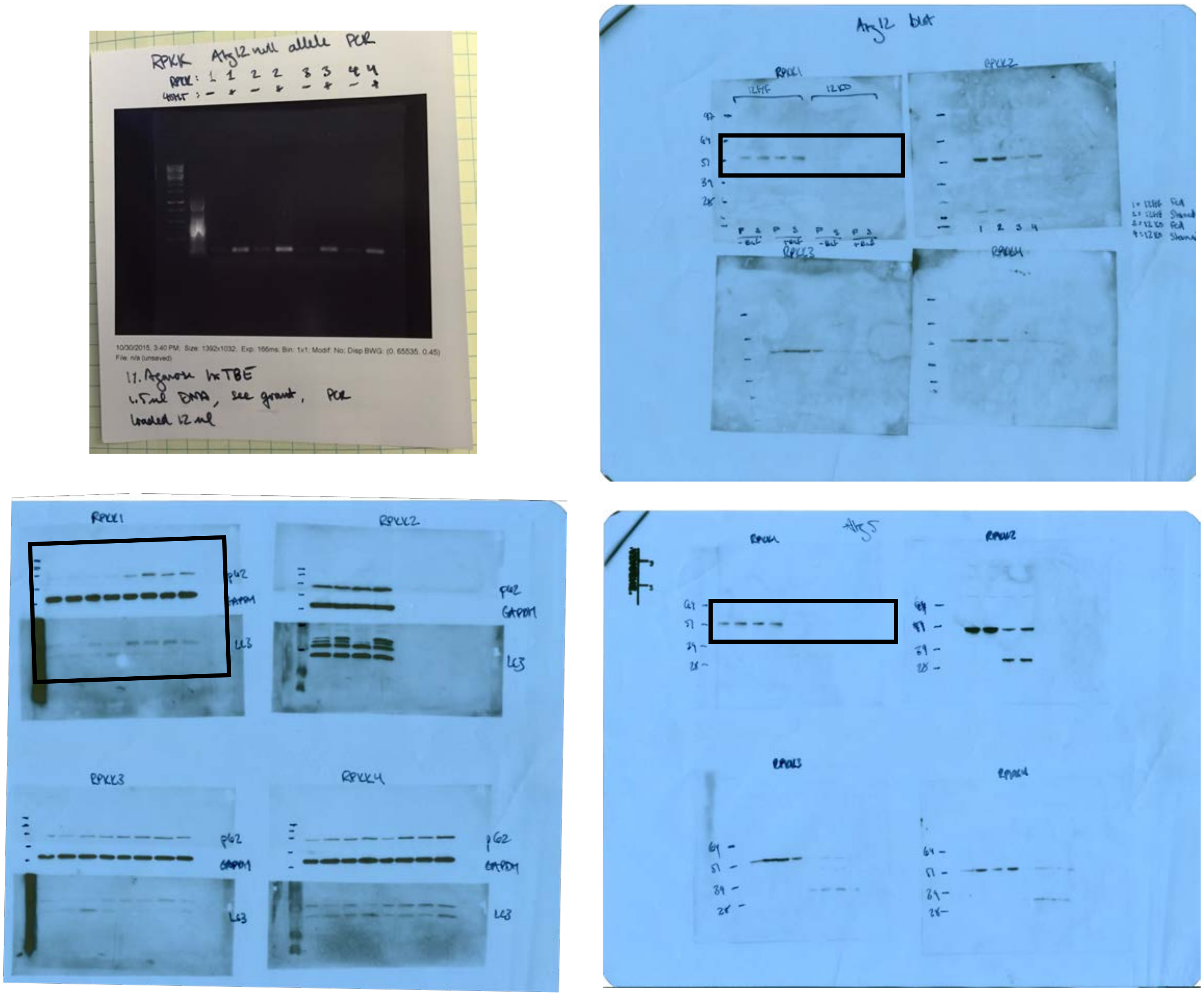

RPKK Atg12 null allele PCR
1% Agarose 1x TBE
Atg12 blot
RPKK1
RPKK2
RPKK3
RPKK4
p62
GAPDH
LC3
Atg5

Figure S1D

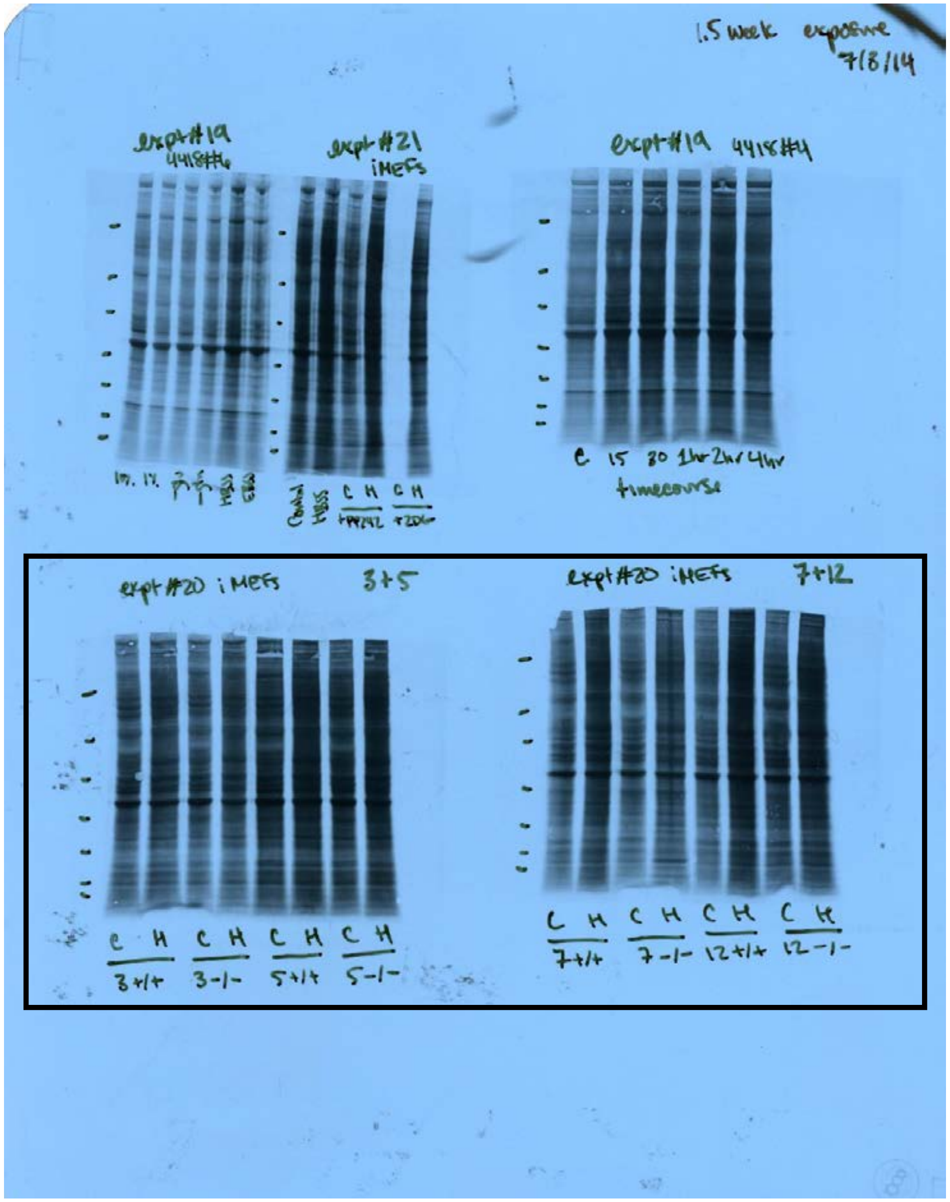
1.5 week exposure
7/8/14
expt #19
4418#6
expt #21
iMEFs
expt #19 4418#4
C 15 30 1hr 2hr 4hr
timecourse
expt #20 iMEFs 3+5
expt #20 iMEFs 7+12
C H C H C H C H
3+/+ 3-/- 5+/+ 5-/-
C H C H C H C H
7+/+ 7-/- 12+/+ 12-/-

Figure S4A

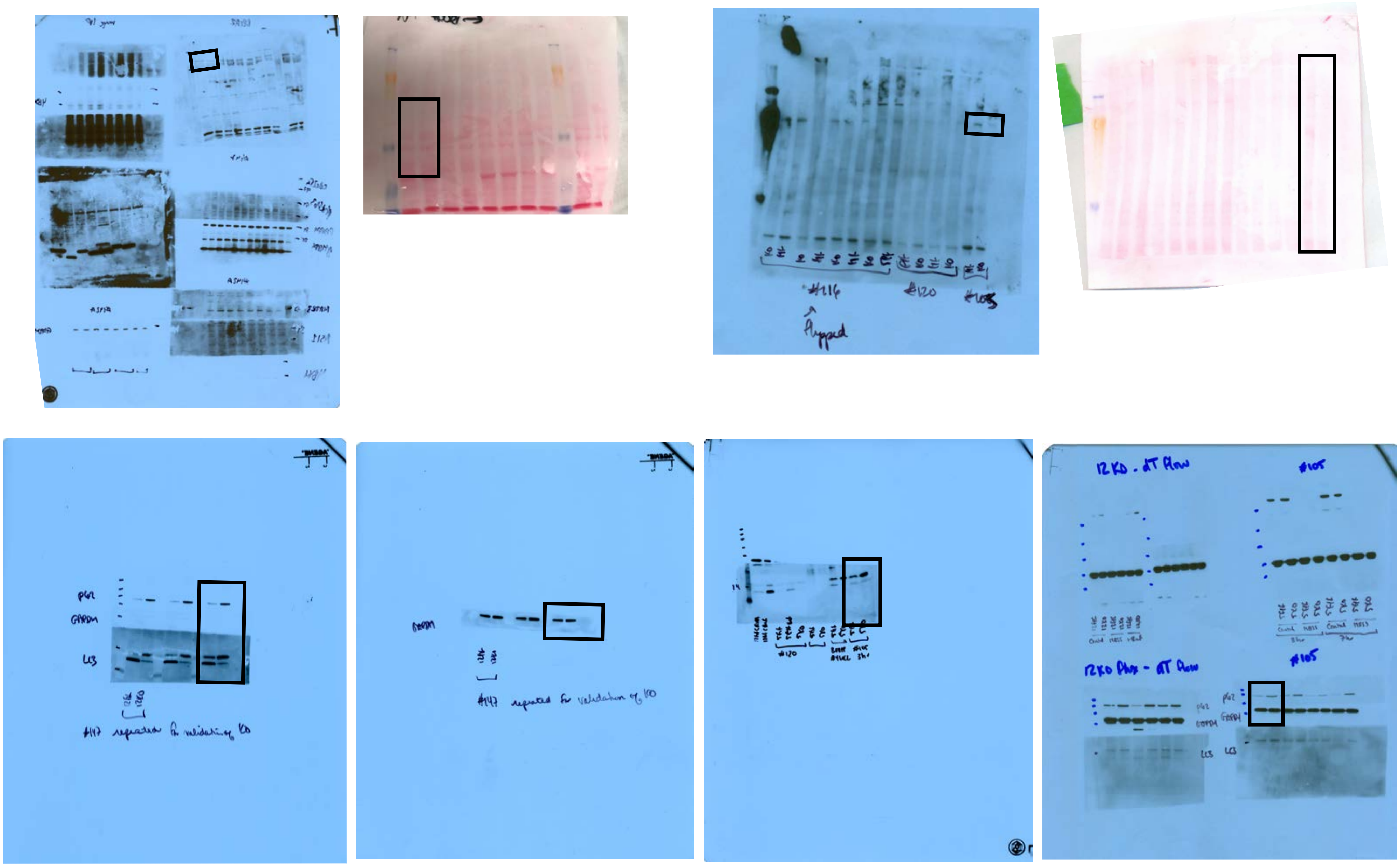

#116
#120
flipped
p62
GAPDH
LC3
#147 repeated for validating KD
#105

Figure S4C

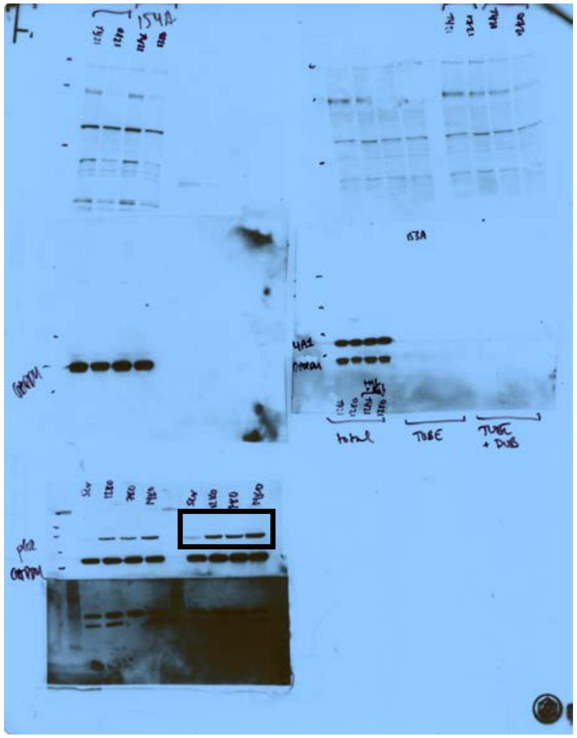

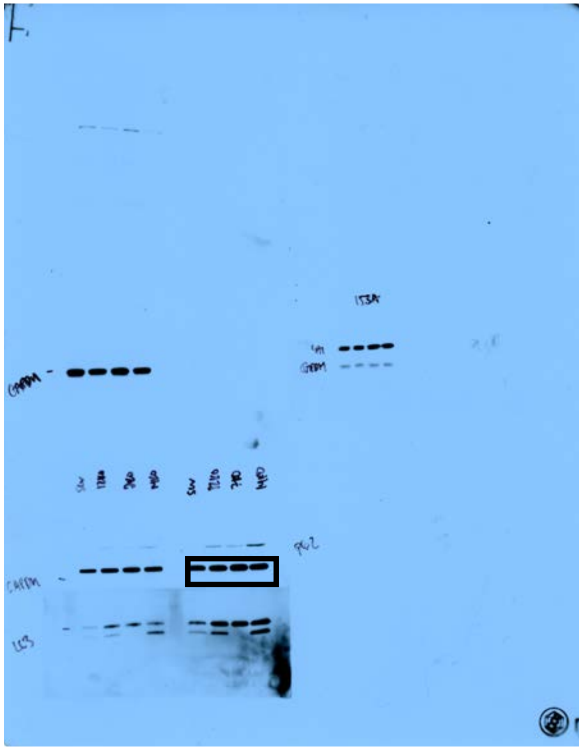

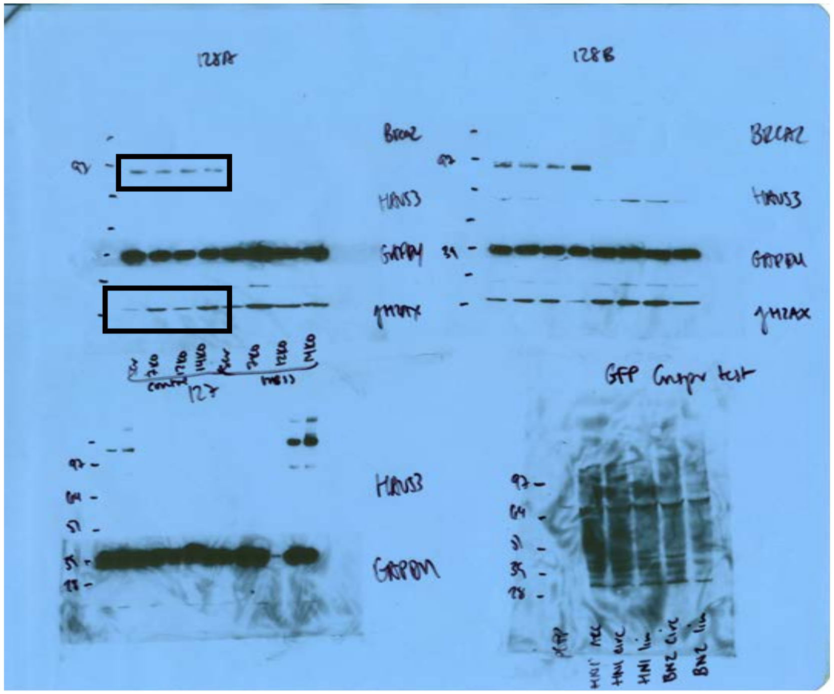

Figure S4E

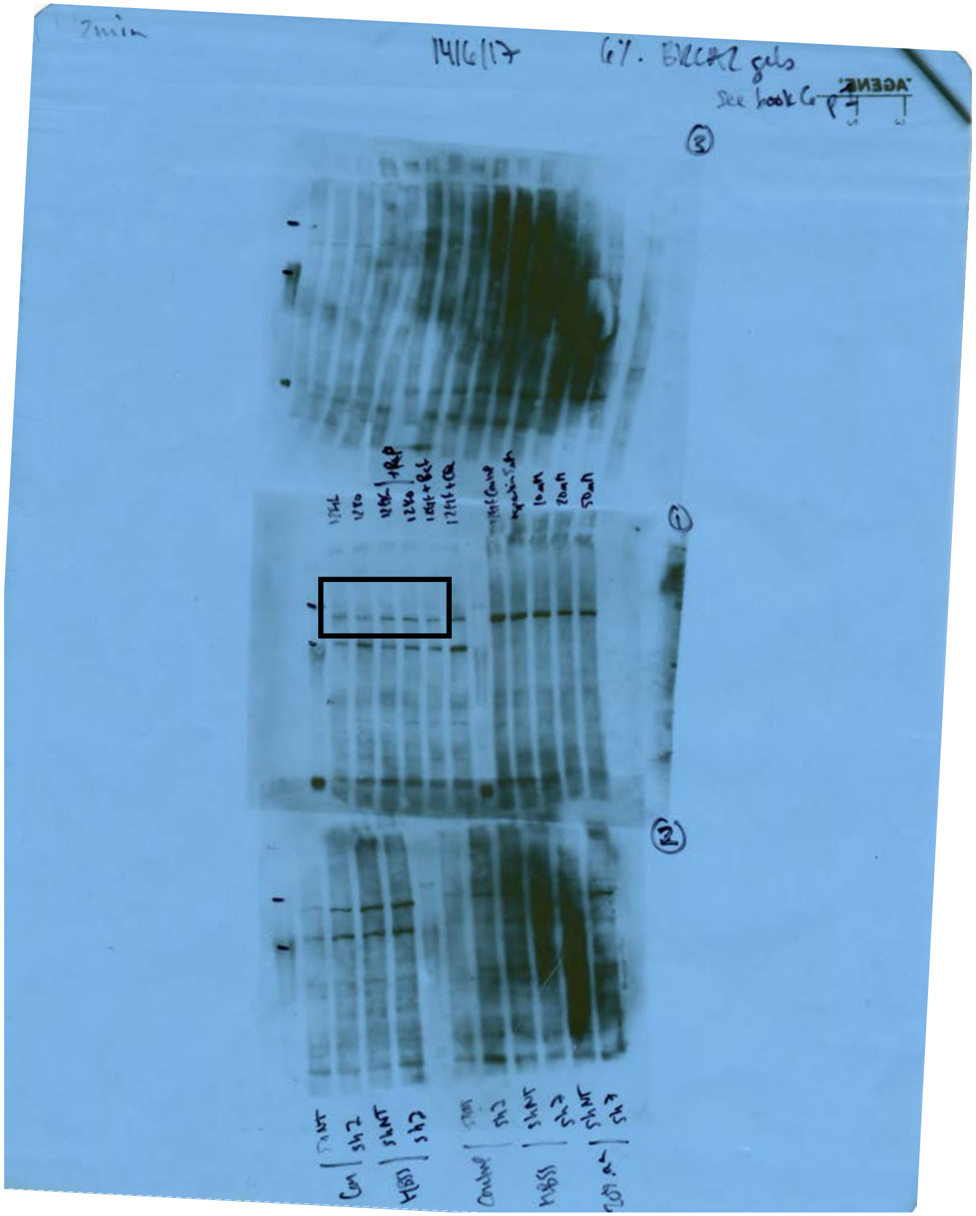

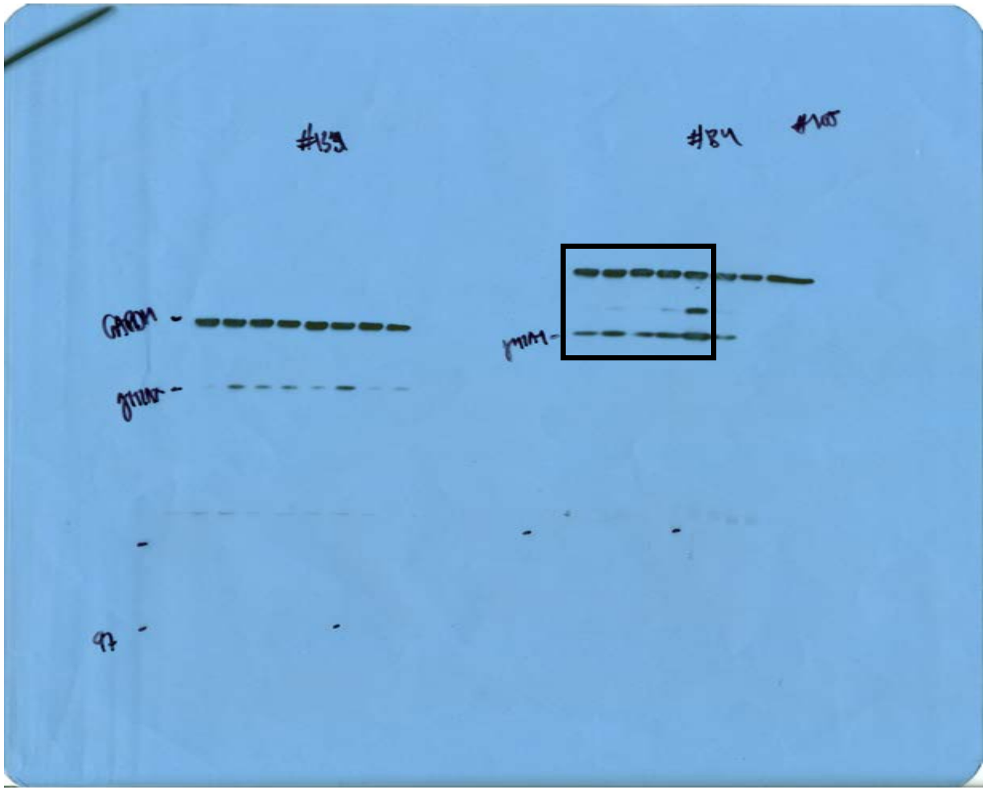

Figure S4G

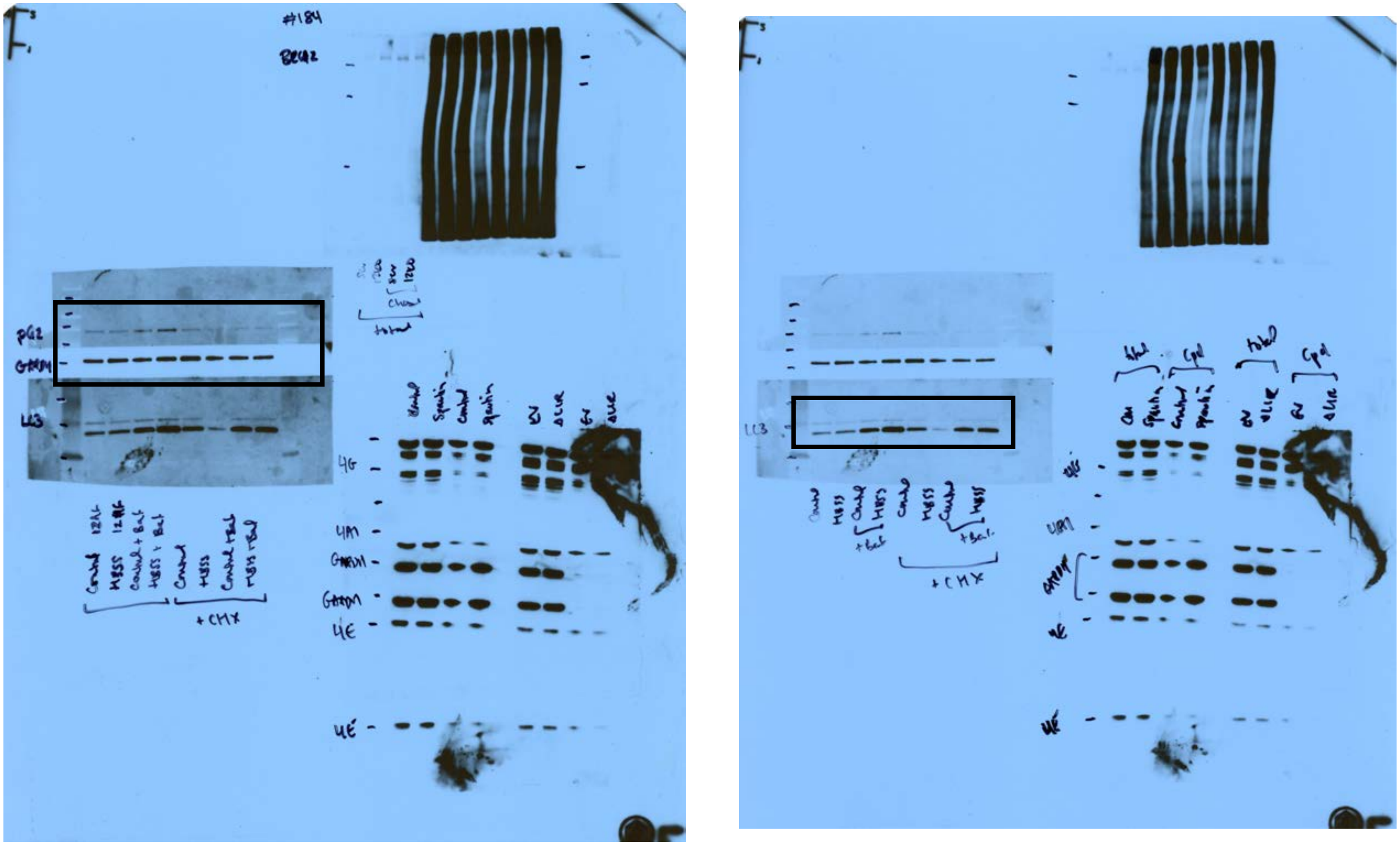

Figure S5E

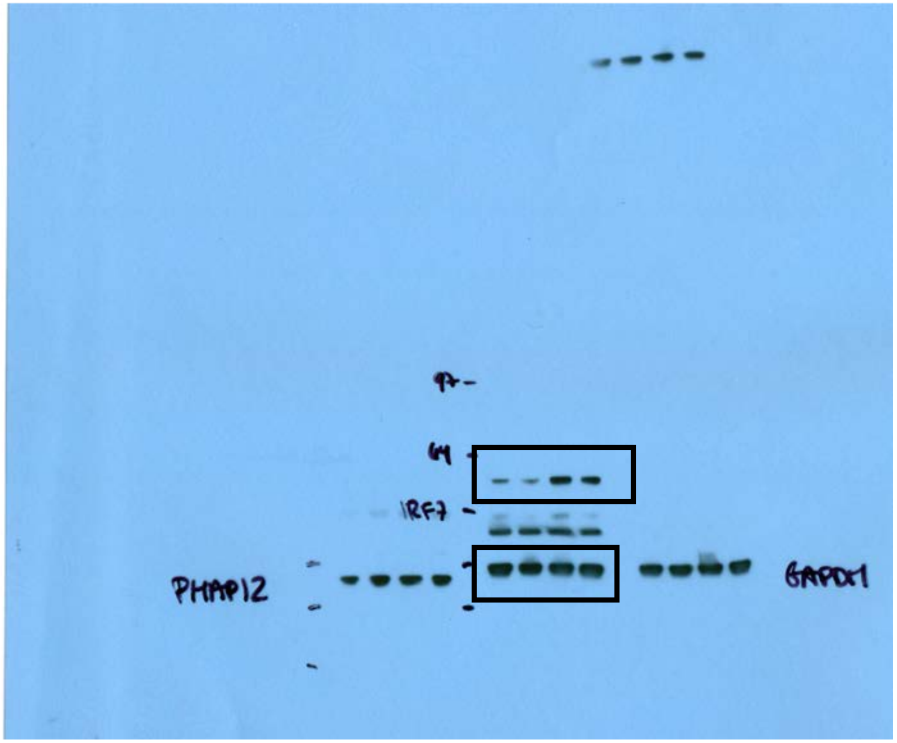

Figure S5F

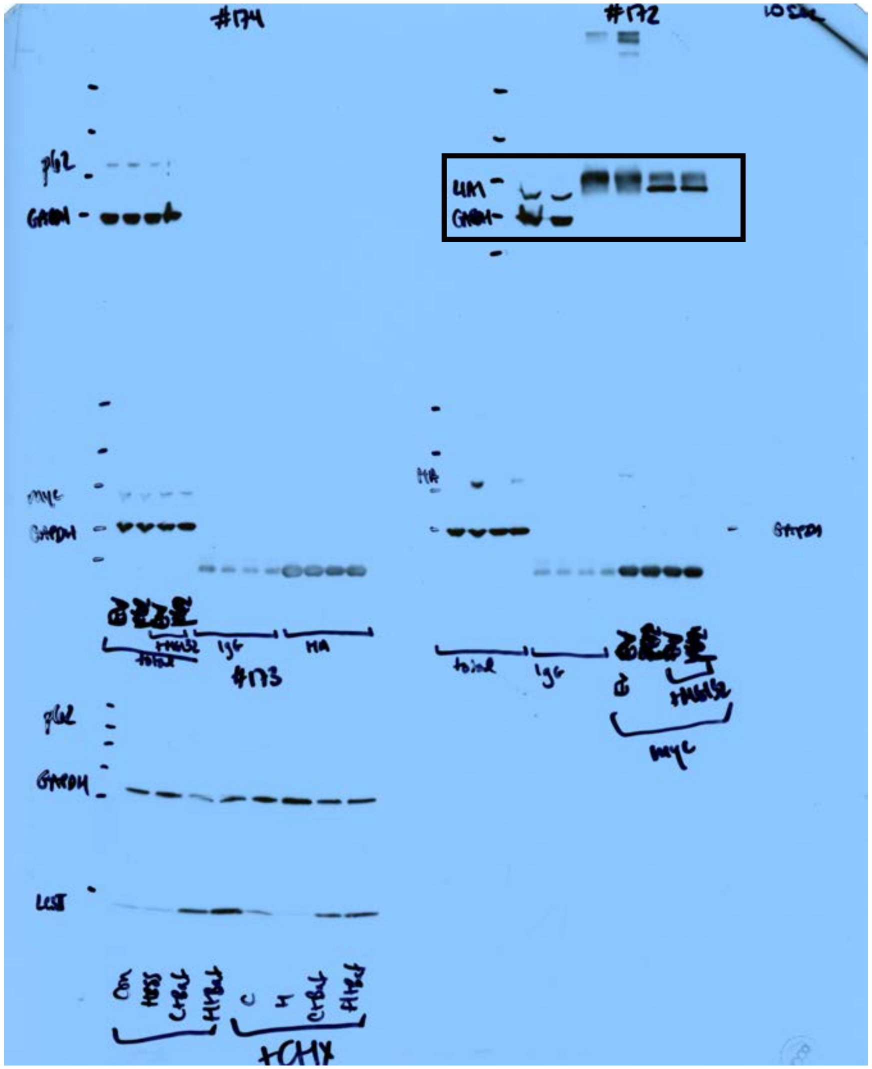

Figure S6A

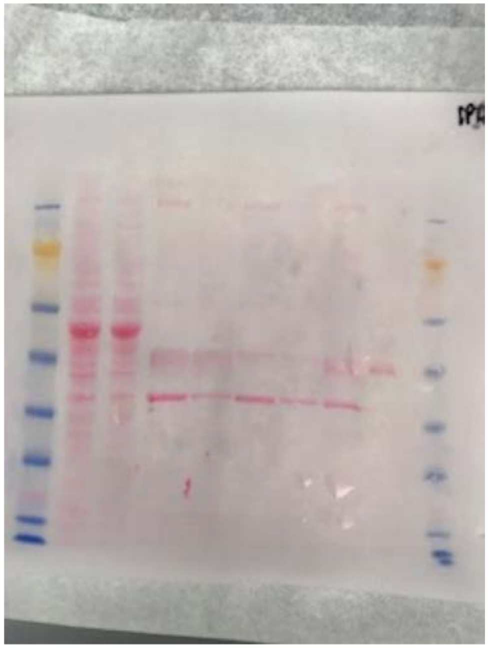

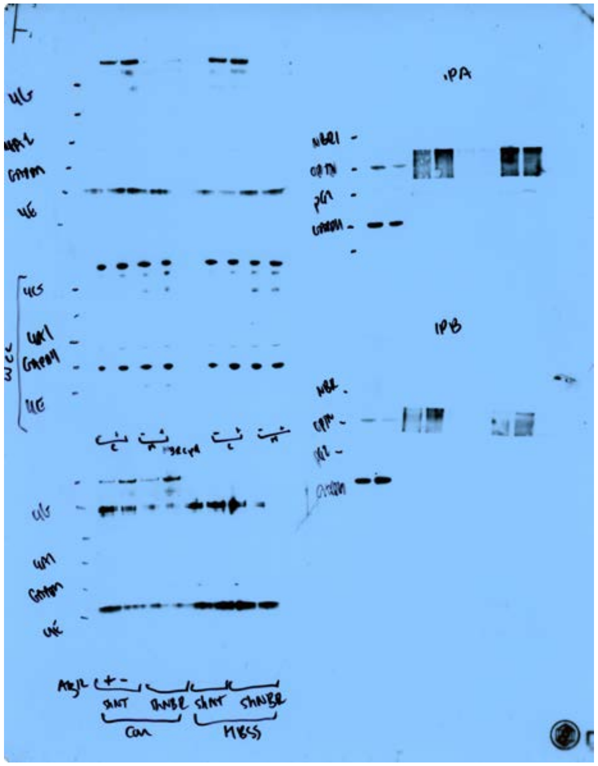
IPA
IPB
4E
Con
HBSS

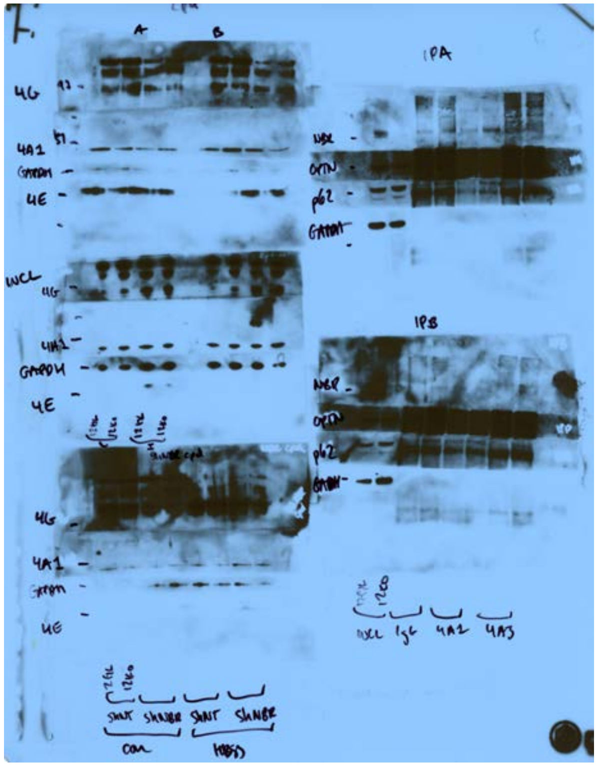
A
B
4G
4A1
4E
IPA
IPB
OPTN
p62
GAPDH
WCL
Con
HBSS

Figure S7D

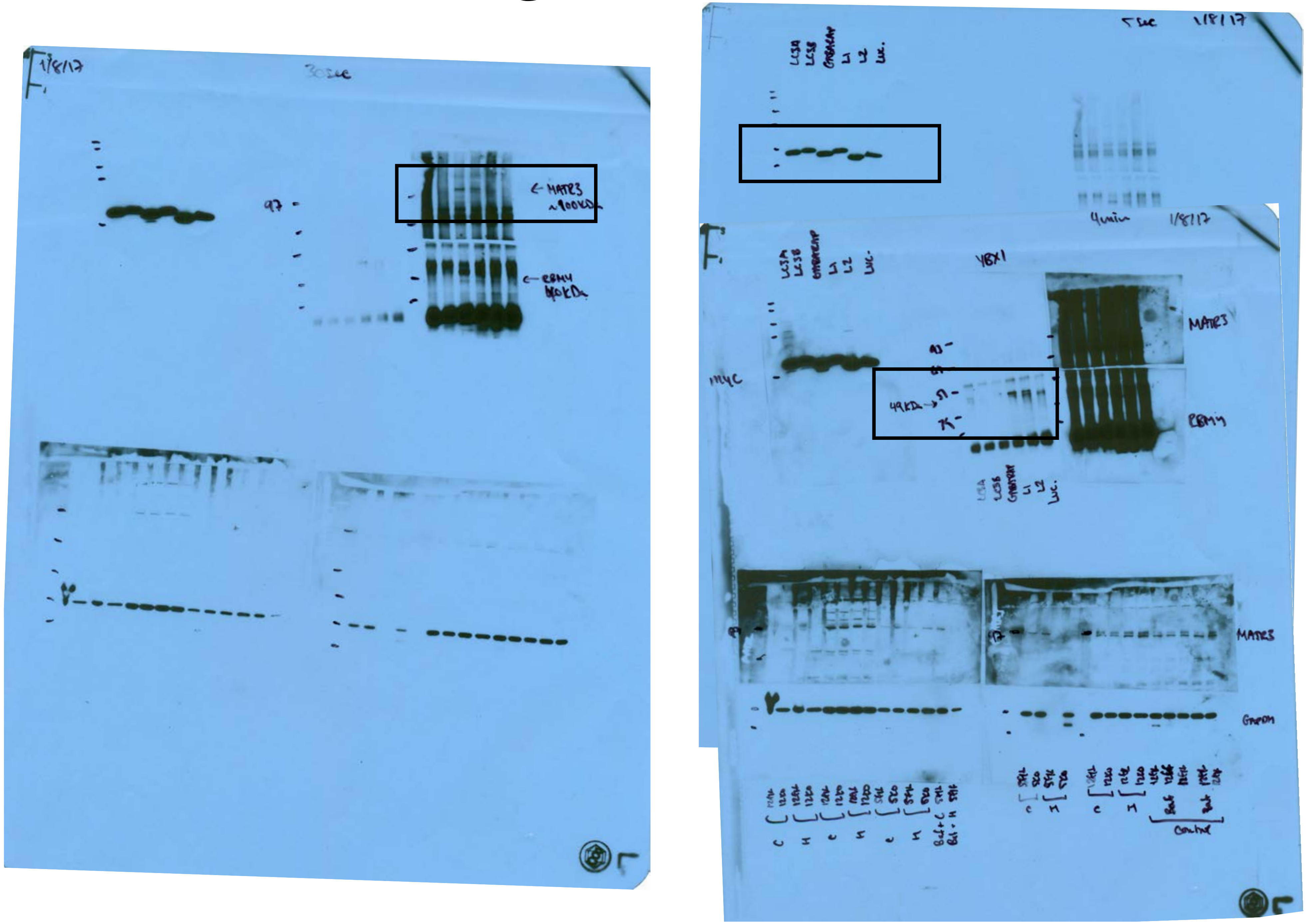
1/8/17
← MATR3
← RBM4
5sec
1/8/17
4min
1/8/17
YBX1
MATR3
RBM4
myc
MATR3
GAPDH

Figure S8A

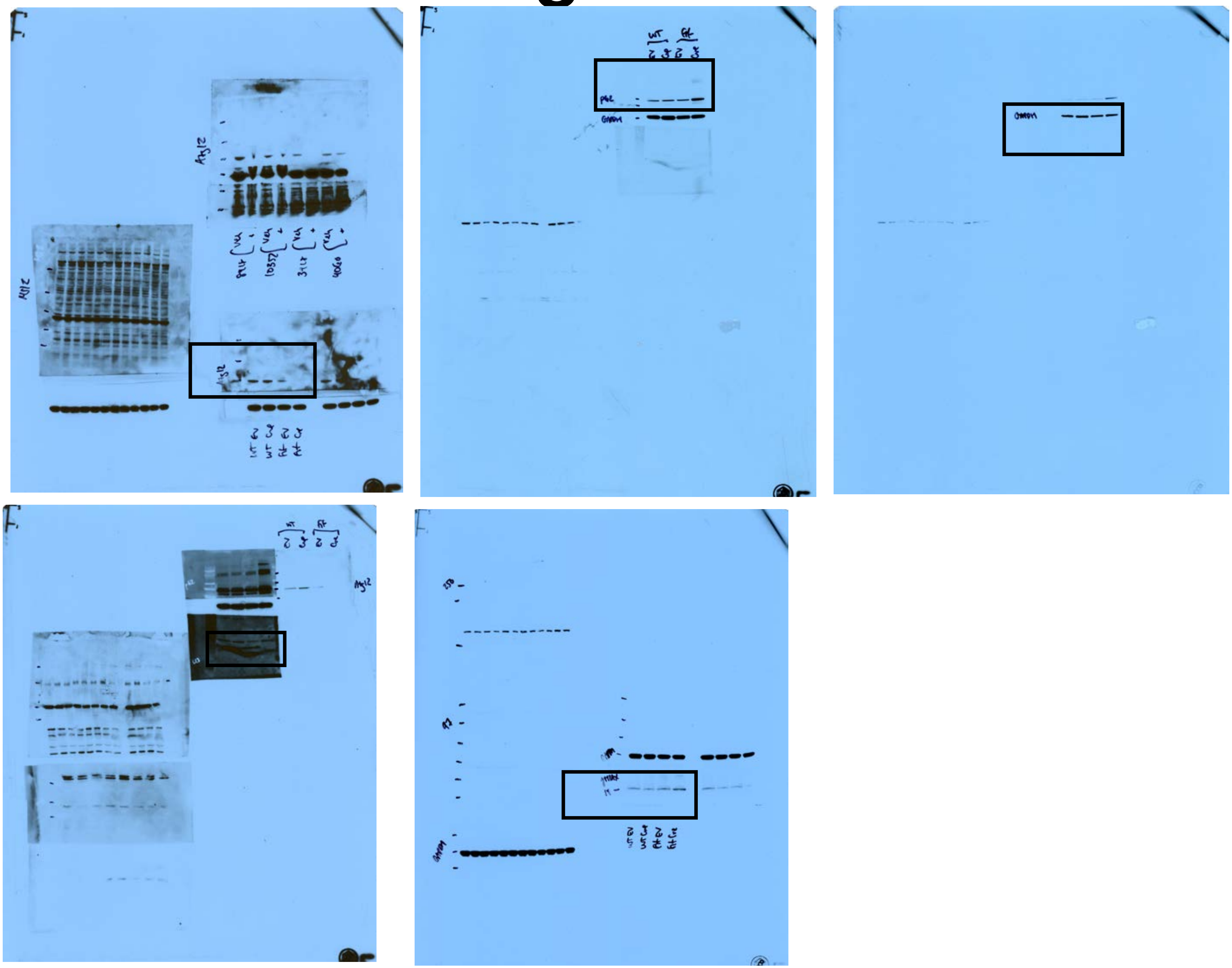

Figure S8D

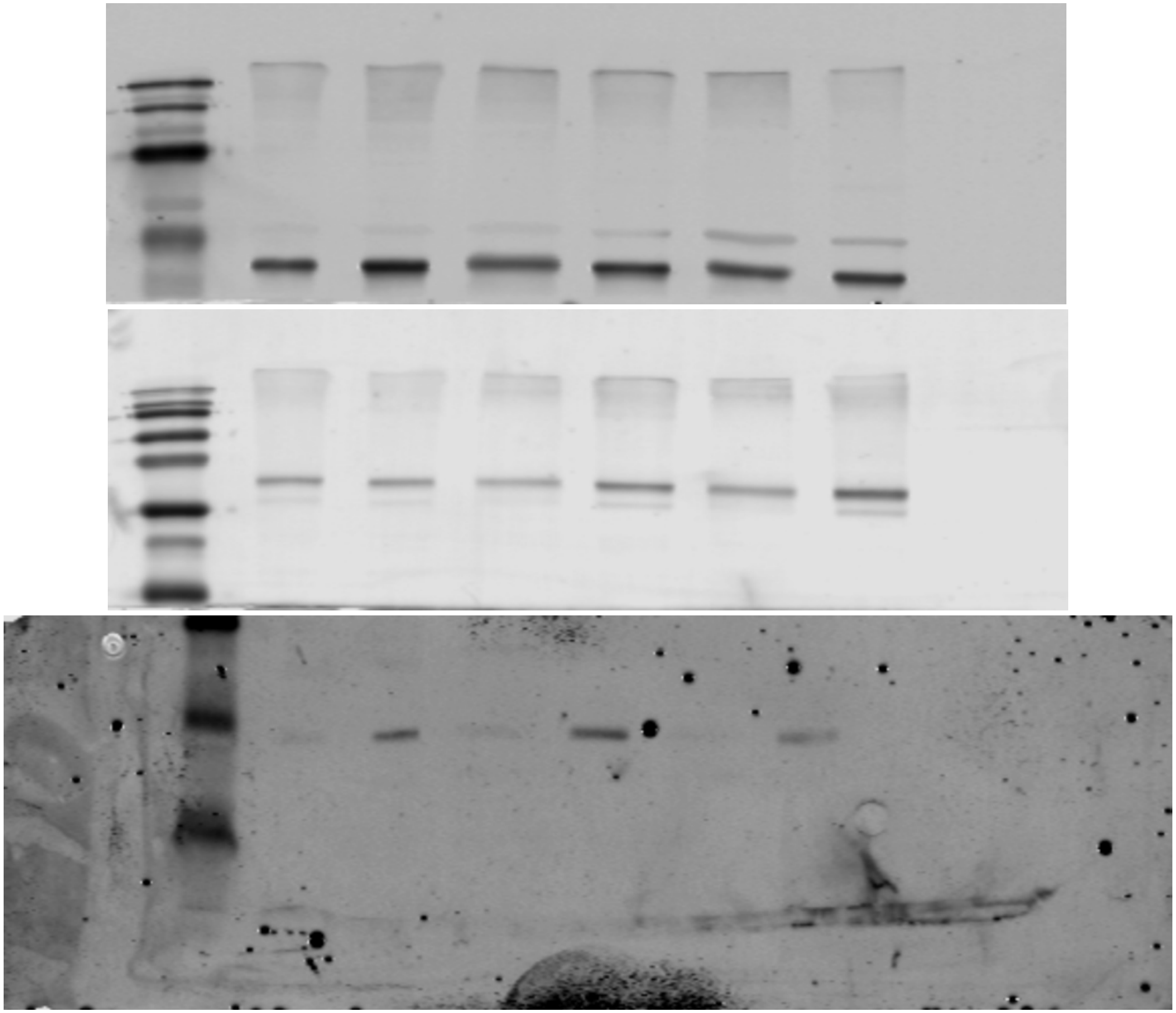

Supplement: Supplementary file 1 — Supplementary Information [file 42003_2020_1090_MOESM1_ESM.pdf]
