# Supplementary material for: Ribosome profiling reveals a functional role for autophagy in mRNA translational control
Source: Commun Biol. 2020 Jul 17;3:388. doi: 10.1038/s42003-020-1090-2 (PMC7367890; doi:10.1038/s42003-020-1090-2)
Supplement: Supplementary file 2 — Description of Additional Supplementary Files [file 42003_2020_1090_MOESM2_ESM.pdf]

Description of Additional Supplementary Files

File Name: Supplementary Data 1
Description: Source data for all plots.

File Name: Supplementary Data 2
Description: Top changes in ribosome occupancy.
