# Supplementary material for: Ribosome profiling reveals a functional role for autophagy in mRNA translational control
Source: Commun Biol. 2020 Jul 17;3:388. doi: 10.1038/s42003-020-1090-2 (PMC7367890; doi:10.1038/s42003-020-1090-2)
Supplement: Supplementary file 6 — Reporting Summary [file 42003_2020_1090_MOESM6_ESM.pdf]

nature research

Corresponding author(s): Debnath

Last updated by author(s): 06/03/2020

Reporting Summary

Nature Research wishes to improve the reproducibility of the work that we publish. This form provides structure for consistency and transparency in reporting. For further information on Nature Research policies, see Authors & Referees and the Editorial Policy Checklist .

Statistics

For all statistical analyses, confirm that the following items are present in the figure legend, table legend, main text, or Methods section.

n/a	Confirmed	
☐	☒	The exact sample size (*n*) for each experimental group/condition, given as a discrete number and unit of measurement
☐	☒	A statement on whether measurements were taken from distinct samples or whether the same sample was measured repeatedly
☐	☒	The statistical test(s) used AND whether they are one- or two-sided<br>*Only common tests should be described solely by name; describe more complex techniques in the Methods section.*
☒	☐	A description of all covariates tested
☐	☒	A description of any assumptions or corrections, such as tests of normality and adjustment for multiple comparisons
☐	☒	A full description of the statistical parameters including central tendency (e.g. means) or other basic estimates (e.g. regression coefficient) AND variation (e.g. standard deviation) or associated estimates of uncertainty (e.g. confidence intervals)
☐	☒	For null hypothesis testing, the test statistic (e.g. *F*, *t*, *r*) with confidence intervals, effect sizes, degrees of freedom and *P* value noted<br>*Give P values as exact values whenever suitable.*
☒	☐	For Bayesian analysis, information on the choice of priors and Markov chain Monte Carlo settings
☒	☐	For hierarchical and complex designs, identification of the appropriate level for tests and full reporting of outcomes
☒	☐	Estimates of effect sizes (e.g. Cohen's *d*, Pearson's *r*), indicating how they were calculated

Our web collection on statistics for biologists contains articles on many of the points above.

Software and code

Policy information about availability of computer code

Data collection	No software was used for data collection.
Data analysis	Ribosome profiling analysis was performed using Babel (Olshen et al, 2013) and all code for subsequent analysis is deposited on Dryad and clearly cited in the paper.

For manuscripts utilizing custom algorithms or software that are central to the research but not yet described in published literature, software must be made available to editors/reviewers. We strongly encourage code deposition in a community repository (e.g. GitHub). See the Nature Research guidelines for submitting code & software for further information.

Data

Policy information about availability of data

All manuscripts must include a data availability statement. This statement should provide the following information, where applicable:

- Accession codes, unique identifiers, or web links for publicly available datasets
- A list of figures that have associated raw data
- A description of any restrictions on data availability

RNA sequencing data is deposited on NCBI with the accession codes included in the publication. All figures have raw data associated in an excel sheet or powerpoint.

Field-specific reporting

Please select the one below that is the best fit for your research. If you are not sure, read the appropriate sections before making your selection.

Life sciences study design

All studies must disclose on these points even when the disclosure is negative.

Sample size	Sample sizes for experiments are consistent with the consensus in the field (eg. minimum of biological triplicate for in vitro experiments), and restricted by reagent availability and feasibility.
Data exclusions	An outlier test was performed on the data, and if a point was found to be statistically significant, that single outlier was excluded from statisticial analysis, but the data point is still shown in the graphs.
Replication	Experiments were repeated a minimum of biological triplicate, with technical replicates where appropriate.
Randomization	Both male and female mice were used at equivalent numbers for comparisons of mouse data.
Blinding	Immunofluorescence image analysis, counting of colonies, were blinded during quantification by a coded sample ID number.

Reporting for specific materials, systems and methods

We require information from authors about some types of materials, experimental systems and methods used in many studies. Here, indicate whether each material, system or method listed is relevant to your study. If you are not sure if a list item applies to your research, read the appropriate section before selecting a response.

Materials & experimental systems

n/a	Involved in the study	
☐	☒	Antibodies
☐	☒	Eukaryotic cell lines
☒	☐	Palaeontology
☐	☒	Animals and other organisms
☒	☐	Human research participants
☒	☐	Clinical data

Methods

n/a	Involved in the study	
☒	☐	ChIP-seq
☒	☐	Flow cytometry
☒	☐	MRI-based neuroimaging

Antibodies

Antibodies used	anti-BRCA2 rabbit pAb from Bioss USA bs1210R; anti-p62 guinea pig pAb from Progen GP-62-C; anti-LC3 mouse pAb, Fung et al 2008 commercially available form EMD Millipore ABC232; anti-phospho-S6 S240/244 rabbit mAb Cell Signaling #2215; anti-S6 rabbit mAb Cell Signaling #2217; anti- α tubulin rabbit mAb Cell Signaling #2125BC; anti-phospho-4EBP1 S65 rabbit pAb Cell Signaling #9451; anti-4EBP1 rabbit pAb Cell Signaling #9452; anti-GAPDH mouse mAb Millipore MAB374; anti-phospho-eIF2α S51 rabbit pAb Cell Signaling #9721; anti- eIF2α S51 rabbit pAb Cell Signaling #9722; anti-eIF4G1 rabbit mAb Cell Signaling #2469; anti-eIF4G2 rabbit pAb Cell Signaling #2182; anti-eIF4E rabbit pAb Cell Signaling #9742; anti-eIF4E2 rabbit pAb Pierce #PA5-11798; anti-Atg12 (mouse specific) rabbit pAb Cell Signaling #2011BC; anti-Atg5 rabbit pAb Novus Biologicals NB110-53818; anti-Atg7 rabbit pAb Cell Signaling #2631; anti-Atf4 rabbit mAb Cell Signaling #11815; anti-Cdc25a rabbit pAb Cell Signaling #3652; anti-Cpt2 Abcam ab71435; anti-Pfkfb3 rabbit pAb ABclonal Biotech A6945; anti-eEF2 rabbit pAb Cell Signaling #2332; anti-Mcl1 rabbit pAb Rockland 800-401-394S; anti-Irf7 rabbit mAb Abcam ab109255; anti-γH2AX S139 mouse mAb Upstate #05-636; anti-cleaved Caspase 3 (Asp175) Rabbit pAb Cell Signaling #9661; anti-eIF4A1 rabbit pAb Cell Signaling #2490; anti-MSI1 rabbit pAb EMD Millipore AB5977; anti-GFP mouse mAb Neuromab N86/8;anti-puromycin mouse mAb Kerafast EQ0001; anti-BRCA2 rabbit pAb Abcam ab123491; anti-MSI1 EMD Millipore AB5977; anti-eIF4A1 Cell Signaling #2490; anti-c-Myc clone 9E10 Sigma-Aldrich M5546; normal rabbit IgG Santa Cruz Biotechnology sc2027; anti-γH2AX S139 Cell Signaling #9718S; anti-53BP1 rabbit pAb Abcam ab21083; anti-γ tubulin mouse mAb Sigma T5326; anti-phospho Histone H3 S10 rabbit pAb Cell Signaling #9701;anti-NBR1 (4BR) mouse mAb Santa Cruz Biotechnology sc1030380
Validation	All antibodies were researched before purchase and selected based on 1) use in previous publications, 2) appropriate species reactivity, 3) validation in our lab or collaborating labs at UCSF, 4) availability at time of purchase. The anti-BRCA2 rabbit pAb from Bioss USA bs1210R has been validated by the independent validation program, details available on the company website:https://www.biossusa.com/products/bs-1210r. the anti-BRCA2 antibody used for IP in human cell lines Abcam ab123491 has been cited, details available on the company website: https://www.abcam.com/brca2-antibody-ab123491-references.html#active-tab. The autophagy related antibodies (anti-p62 guinea pig pAb from Progen GP-62-C; anti-LC3 mouse pAb, Fung et al 2008 commercially available form EMD Millipore ABC232; anti-Atg12 (mouse specific) rabbit pAb Cell Signaling #2011BC; anti-Atg5 rabbit pAb Novus Biologicals NB110-53818; anti-Atg7 rabbit pAb Cell Signaling #2631) have been previously validated by our lab and used in a number of previous publications. Loading control antibodies and antibodies to tags (anti-GAPDH MAB374; anti- α tubulin CST #2125BC; anti-GFP mouse mAb Neuromab N86/8; anti-c-Myc clone 9E10 Sigma-Aldrich M5546) are commonly used and published antibodies, details on company website: http://www.emdmillipore.com/US/en/product/Anti-Glyceraldehyde-3-Phosphate-Dehydrogenase-Antibody-clone-6C5,MM_NF-MAB374#anchor_REF, https://www.cellsignal.com/products/primary-antibodies/a-tubulin-11h10-rabbit-mab/2125?_=1560381523571&Ntt=2125&tahead=true, http://neuromab.ucdavis.edu/datasheet/N86_8.pdf, https://www.sigmaaldrich.com/catalog/product/mm/op10?lang=en&region=US. Antibodies downstream of mTOR signaling (anti-phospho-4EBP1 S65 rabbit pAb Cell Signaling #9451; anti-4EBP1 rabbit pAb Cell Signaling #9452; anti-phospho-S6 S240/244

rabbit mAb Cell Signaling #2215; anti-S6 rabbit mAb Cell Signaling #2217) are heavily used in the literature and well cited, details on the cell signaling website:https://www.cellsignal.com/. Initiation factor antibodies (anti-phospho-eIF2α S51 rabbit pAb Cell Signaling #9721; anti- eIF2α S51 rabbit pAb Cell Signaling #9722; anti-eIF4G1 rabbit mAb Cell Signaling #2469; anti-eIF4G2 rabbit pAb Cell Signaling #2182; anti-eIF4E rabbit pAb Cell Signaling #9742; anti-eEF2 rabbit pAb Cell Signaling #2332; anti-eIF4A1 rabbit pAb Cell Signaling #2490) have been used extensively in the literature, described on the company website: https://www.cellsignal.com. Antibodies used to validate translational changes measured by ribosome profiling (anti-Atf4 rabbit mAb Cell Signaling #11815; anti-Cdc25a rabbit pAb Cell Signaling #3652; anti-Cpt2 Abcam ab71435) have been well cited in the literature, details available on company websites:https://www.cellsignal.com, https://www.abcam.com/cpt2cpt1-antibody-ab71435.html. anti-MSI1 rabbit pAb EMD Millipore AB5977 was validated in the study in combination with shRNAs purchased from Sigma, that are not predicted to target another mRNA. anti-γH2AX S139 mouse mAb Upstate #05-636, references on the company website: http://www.emdmillipore.com/US/en/product/Anti-phospho-Histone-H2A.X-Ser139-Antibody-clone-JBW301,MM_NF-05-636#documentation. anti-cleaved Caspase 3 (Asp175) Rabbit pAb Cell Signaling #9661 references on the company website: https://www.cellsignal.com/products/primary-antibodies/cleaved-caspase-3-asp175-antibody/9661.

Eukaryotic cell lines

Policy information about cell lines

Cell line source(s)	N. Mizushima (University of Tokyo, Japan) provided Atg5+/+, Atg5−/−, Atg7+/+ and Atg7-/- MEFs and M. Komatsu (Tokyo Metropolitan Institute, Japan) provided Atg3+/+ and Atg3−/− MEFs. Atg12+/+ and Atg12-/- MEFs were originally generated in Malhotra et al, 201510. HEK293Ts were cultured in DMEM 1x (Gibco) supplemented with 10% FBS (Atlas) and Pen/Strep. HEK293Ts were purchased from ATCC (CRL-3216).
Authentication	HEK293Ts were purchased from ATCC, used within 20 passages, and authenticated using STR profiling. MEFs were authenticated via PCR genotyping.
Mycoplasma contamination	Cell lines were routinely tested for mycoplasma. The creation of MEF cell lines were treated prior to any experimentation with plasmocin according to manufacturers instructions so that mycoplasma tests were negative.
Commonly misidentified lines (See ICLAC register)	The study did not use any commonly misidentified cell lines.

Animals and other organisms

Policy information about studies involving animals; ARRIVE guidelines recommended for reporting animal research

Laboratory animals	Compound transgenic C57Bl/6 mice harboring Atg12f/f and Cag-CreER were generated by cross-breeding of Atg12f/f 10 and CagCreER animals (obtained via the UCSF mouse database). Both male and female mice at 16 weeks of age were used.
Wild animals	The study did not involve wild animals.
Field-collected samples	The study did not involve field-collected samples
Ethics oversight	All experimental procedures and treatments were conducted in compliance with UCSF Institutional Animal Care and Use Committee guidelines.

Note that full information on the approval of the study protocol must also be provided in the manuscript.
